# *Clostridium perfringens* phospholipase C, an archetypal bacterial virulence factor, induces the formation of extracellular traps by human neutrophils

**DOI:** 10.3389/fcimb.2023.1278718

**Published:** 2023-10-27

**Authors:** Lisa Badilla-Vargas, Reynaldo Pereira, José Arturo Molina-Mora, Alberto Alape-Girón, Marietta Flores-Díaz

**Affiliations:** ^1^ Instituto Clodomiro Picado, Facultad de Microbiología, Universidad de Costa Rica, San José, Costa Rica; ^2^ Departamento de Bioquímica, Escuela de Medicina, Universidad de Costa Rica, San José, Costa Rica; ^3^ Centro Nacional de alta Tecnología, Consejo Nacional de Rectores (CONARE), San José, Costa Rica; ^4^ Centro de investigación en Enfermedades Tropicales, Facultad de Microbiología, Universidad de Costa Rica, San José, Costa Rica

**Keywords:** *Clostridium perfringens*, secretome, bacterial toxins, phospholipase, bacterial pathogenesis, NETs, antioxidants, gas gangrene

## Abstract

Neutrophil extracellular traps (NETs) are networks of DNA and various microbicidal proteins released to kill invading microorganisms and prevent their dissemination. However, a NETs excess is detrimental to the host and involved in the pathogenesis of various inflammatory and immunothrombotic diseases. *Clostridium perfringens* is a widely distributed pathogen associated with several animal and human diseases, that produces many exotoxins, including the phospholipase C (CpPLC), the main virulence factor in gas gangrene. During this disease, CpPLC generates the formation of neutrophil/platelet aggregates within the vasculature, favoring an anaerobic environment for *C. perfringens* growth. This work demonstrates that CpPLC induces NETosis in human neutrophils. Antibodies against CpPLC completely abrogate the NETosis-inducing activity of recombinant CpPLC and *C. perfringens* secretome. CpPLC induces suicidal NETosis through a mechanism that requires calcium release from inositol trisphosphate receptor (IP_3_) sensitive stores, activation of protein kinase C (PKC), and the mitogen-activated protein kinase/extracellular signal-regulated kinase (MEK/ERK) pathways, as well as the production of reactive oxygen species (ROS) by the metabolism of arachidonic acid. Proteomic analysis of the *C. perfringens* secretome identified 40 proteins, including a DNAse and two 5´-nucleotidases homologous to virulence factors that could be relevant in evading NETs. We suggested that in gas gangrene this pathogen benefits from having access to the metabolic resources of the tissue injured by a dysregulated intravascular NETosis and then escapes and spreads to deeper tissues. Understanding the role of NETs in gas gangrene could help develop novel therapeutic strategies to reduce mortality, improve muscle regeneration, and prevent deleterious patient outcomes.

## Introduction

Neutrophils play a critical role in the innate immune response against pathogens through chemokines and cytokines secretion, degranulation, phagocytosis, ROS generation, and the release of NETs (Liew and Kubes, 2019). NETs are filamentous structures of decondensed DNA coated with several proteins with microbicidal activity that trap and kill pathogens, preventing their dissemination (Papayannopoulos, 2018; Schultz et al., 2022). However, NETs could exacerbate inflammation and activate the coagulation system (de Bont et al., 2019; Morales-Primo et al., 2022). Therefore, NETs formation should be highly regulated and balanced with their degradation by endogenous DNAses and macrophages to avoid host tissue damage (Demkow, 2023; Poli and Zanoni, 2023). Indeed, an excess of NETs is detrimental to the host and is involved in the pathogenesis of various inflammatory and immunothrombotic diseases (Apel et al., 2018; Zhu et al., 2022a; Zhu et al., 2022b).

NETosis is an ancient and evolutionary conserved defense mechanism induced by microorganisms such as parasites, fungi, and viruses (Schultz et al., 2022; Poli and Zanoni, 2023). In addition, NETs formation could also be induced and/or boosted by proinflammatory cytokines, such as TNF-α, IL1β, IL-8, and IL-12, as well as other endogenous stimuli that include immune complexes, activated complement proteins, and platelets (Zucoloto and Jenne, 2019; Poli and Zanoni, 2023). Indeed, NETs and platelets constitute an intricate network that interweaves inflammation with blood coagulation (Wienkamp et al., 2022). Thus, during certain infections, intravascular NETs could lead to immune-mediated thrombus formation in microvessels that facilitate pathogen containment and destruction (Zucoloto and Jenne, 2019). In some pathologies, such as sepsis and COVID-19, an excess of intravascular NETs damages the endothelium and drives thrombosis, leading to blood flow occlusion and injury of the host tissues (Zhu et al., 2022a; Zhu et al., 2022b). In other infections, the pathogen produces virulence factors to modulate NETs formation or evade their entrapment, such as DNAses, which break down the integrity of the NET-backbone (Von Köckritz-Blickwede et al., 2016; Ríos-López et al., 2021; de Jesus Gonzalez-Contreras and Zarate, 2022; Janssen et al., 2022).

NETosis is classified into suicidal and vital NETosis (Thiam et al., 2020; Singh et al., 2023). In both cases, an increase in intracellular calcium levels and ROS activate several downstream effectors crucial for NETs formation (Thiam et al., 2020; Singh et al., 2023). In canonical suicidal NETosis, calcium is released from the endoplasmic reticulum after stimulation, and its increased cytosolic concentration activates PKC, which leads to NADPH oxidase (NOX) assembly (Thiam et al., 2020; Rosazza et al., 2021; Singh et al., 2023). Once NOX is activated, ROS are produced, which stimulates the degradation of cytoplasmic granules and release of myeloperoxidase (MPO), plus azurophilic proteases [azurodicin, cathepsin G, and neutrophil elastase (NE)] (Rosazza et al., 2021; Singh et al., 2023). NE moves into the nucleus, cleaving the N-terminal tails of core histones, which favors chromatin decondensation (Rosazza et al., 2021; Singh et al., 2023). Meanwhile, peptidyl arginine deiminase 4 (PAD4) is also activated, promoting histone H3 citrullination, which is required for chromatin decondensation and DNA extranuclear extrusion (Rosazza et al., 2021; Singh et al., 2023). Disassembly of the cortical cytoskeleton weakens the plasma membrane, which ruptures, releasing NETs into the extracellular space. Thus, neutrophils die after suicidal NETosis, which usually lasts 3-4 h (Thiam et al., 2020; Rosazza et al., 2021; Singh et al., 2023). Vital NETosis is a NOX-independent process induced by some microorganisms, activated platelets, and complement proteins (Thiam et al., 2020; Rosazza et al., 2021; Singh et al., 2023). Calcium ions influx into the cytosol via small conductance potassium channel member three (SK3), activates PAD4, and promotes histone H3 citrullination (Thiam et al., 2020; Rosazza et al., 2021; Singh et al., 2023). The decondensed chromatin, histones, and granular proteins are packed in vesicles that release their content out of the neutrophil upon fusion with the plasma membrane. In vital NETosis, which takes about 30 min, the plasma membrane remains intact, and the neutrophil stays functional after the process (Thiam et al., 2020; Rosazza et al., 2021; Singh et al., 2023).

*C. perfringens* is a Gram-positive, anaerobic, sporulated, nonmotile bacillus widely distributed in the environment and the gastrointestinal tract of humans and animals (Kiu and Hall, 2018). If the environment is suitable, this bacterium can proliferate and produce virulence factors, causing many diseases in humans, poultry, goats, ovines, and bovines (Kiu and Hall, 2018).

*C. perfringens* can produce more than 17 toxins and different extracellular enzymes, including the toxins α, β, ϵ, ι, δ, θ, κ, λ, μ, ν, an enterotoxin (CPE), necrotic enteritis toxin B (NetB), a hemolysin, and neuraminidase (Rood et al., 2018; Mehdizadeh Gohari et al., 2021). Therefore, isolates of this bacterium are classified into seven toxinotypes ranging from A to G, based on their ability to produce different toxins (Rood et al., 2018). Toxinotype A, which has the highest production of CpPLC (or α-toxin), is associated with a human necrotizing soft tissue infection referred to as gas gangrene or clostridial myonecrosis (Rood et al., 2018).

Gas gangrene is an acute and rapidly progressive infection characterized by thrombosis and severe myonecrosis, with the absence of neutrophils in the infected tissue (Stevens and Bryant, 2017). CpPLC is the main virulence factor for *C. perfringens* pathogenicity during gas gangrene (Reviewed in Monturiol-Gross et al., 2021). This toxin favors the production of IL-8 and TNF-α and affects signal transduction pathways in platelets, neutrophils, and endothelial cells, leading to the overexpression of adhesion molecules that alter neutrophil diapedesis (Bryant et al., 2006). CpPLC also causes oxidative stress in the infected tissues in a murine gas gangrene model (Monturiol-Gross et al., 2012). This toxin stimulates phospholipase A_2_ mediated arachidonic acid release and induces ROS production by activating different intracellular signaling pathways involving PKC, ERK/MEK, and the transcription factor NFκB (Gustafson and Tagesson, 1990; Monturiol-Gross et al., 2014). When injected intramuscularly, CpPLC reproduces the physiopathological effects of a *C. perfringens* infection, including extensive myonecrosis, partially preventable by antioxidants (Monturiol-Gross et al., 2012).

*C. perfringens* induces NETs formation in murine neutrophils, which requires the intracellular pore-forming Mixed lineage kinase-like protein that oligomerizes in the membrane (Liu et al., 2023a; Liu et al., 2023b). However, neither the signaling pathway involved in NET induction nor the *C. perfringens* virulence factor responsible for the NET inducing activity have been identified.

Herein, we show for the first time that CpPLC induces NETs formation by human neutrophils, which reveals an unknown effect of bacterial phospholipases as virulence factors. The type of NETosis and the molecular mechanism of NETs formation activated by the CpPLC were characterized, providing novel mechanistic insights for further studies on the molecular pathogenesis of *C. perfringens* infections.

## Materials and methods

### *C. perfringens* secreted proteins

*C. perfringens* JIR325, a toxinotype A strain, generated by spontaneous mutation of strain 13 (Lyristis et al., 1994), was grown in Reinforced Clostridial Medium (RCM). In anaerobiosis at 37°C, a fresh culture was inoculated into RCM until an optical density at 600 nm of 0.4.-0.5 was reached. This was then used as seed culture in a 50 ml flask, which was incubated for 24 h at 37°C. This stationary phase culture was then centrifuged at 1000 x g for 7 min, and the supernatant was filtered through a 0.22 µm pore size membrane. Subsequently, the proteins were concentrated to 5 ml through ultrafiltration using a 10 kDa exclusion membrane, the final protein concentration was 6.3 mg/ml. Total protein concentration was determined using a nanodrop spectrophotometer, and the preparations were stored at -20°C.

### Proteomic analysis

The secreted proteins from the *C. perfringens* stationary phase culture were separated by SDS-PAGE under reducing conditions, and the gel was stained with Coomassie blue. Bands were excised from the gel, and protein disulfide bonds reduced with 10 mM dithiothreitol, followed by alkylation with 50 mM iodoacetamide and overnight in-gel digestion with sequencing grade bovine trypsin (in 25 mM ammonium bicarbonate) using an automated workstation (Intavis). The resulting peptides were dried, redissolved in water with 0.1% formic acid, and submitted to nESI-MS/MS on a Q-Exactive Plus^®^ mass spectrometer (Thermo-Fisher) as previously described (Lomonte and Fernández, 2022). Each tryptic digest (10 µl) was loaded on a C18 trap column (75 μm × 2 cm, 3 μm particle; PepMap^®^, Thermo), washed with 0.1% formic acid, and separated at 200 nl/min with a 3 μm particle, 15 cm × 75 μm C18 Easy-spray^®^ analytical column using a nano-Easy^®^ 1200 chromatograph (Thermo-Fisher). A gradient from 0.1% formic acid (solution A) to 80% acetonitrile with 0.1% formic acid (solution B) was developed: 1–5% B in 1 min, 5–26% B in 25 min, 26–79% B in 4 min, 79–99% B in 1 min, and 99% B in 4 min, for a total time of 35 min. MS spectra were acquired in positive mode at 1.9 kV, with a capillary temperature of 200°C, using 1 μscan at 400–1600 m/z, maximum injection time of 100 msec, AGC target of 3×10^6^, and orbitrap resolution of 70,000. The top 10 ions with 2–5 positive charges were fragmented with AGC target of 1×10^5^, maximum injection time of 110 ms, resolution of 17,500, loop count of 10, isolation window of 1.4 m/z, and a dynamic exclusion time of 5 s. MS/MS spectra were processed for peptide matching against *C. perfringens* protein sequences contained in the UniProt database or against the sequences of seven clostridial nucleases in a private database using Peaks X^®^ (Bioinformatics Solutions) (Tran et al., 2019). Cysteine carbamidomethylation was set as a fixed modification, while deamidation of asparagine or glutamine and methionine oxidation were set as variable modifications, allowing up to 3 missed cleavages by trypsin. Parameters for match acceptance were set to False Discovery Rate <1% -10lgP protein score ≥30.

### Human neutrophils isolation

Human neutrophils were purified from healthy volunteers, as described previously (Brinkmann et al., 2010) with some modifications. Briefly, peripheral blood was collected in tubes containing sodium citrate and then 6 ml of blood was carefully placed on top of 6 ml of Histopaque ^®^-1119 (Sigma-Aldrich, USA). After centrifugation for 20 min at 400 x g without braking, the layer containing the granulocytes was recovered and washed with 10 ml of sterile phosphate buffer solution (PBS). Afterward, the cells were centrifuged at 400 x g for 10 min and the supernatant was discarded. Erythrocyte remnants were removed using 5 ml of lysis buffer (0.155 M NH_4_Cl, 10 mM KHCO_3_, 5% EDTA, and deionized water) for 25 s. Then, 10 ml of sterile PBS was added to stop lysis, and the cell suspension was centrifuged at 400 x g for 10 min. The cell pellet was resuspended in 1 ml of sterile Roswell Park Memorial Institute (RPMI) medium (Sigma-Aldrich) supplemented with 10% fetal bovine serum (inactivated at 56°C) and 1% penicillin-streptomycin. All centrifugation steps were carried out at room temperature (r.t).

### NETs induction and detection by immunofluorescence

NETs induction of human neutrophils was carried out as previously described (Brinkmann et al., 2010). A 24-well plate was prepared with sterile glass coverslips (10 mm diameter) previously coated with 0.001% poly-L-lysine for 1 h. Then, freshly isolated neutrophils were seeded at 4x10^5^ per well in 500 μl of RPMI medium and incubated for 1 h at 37°C in a humid atmosphere with 7% CO_2_ to allow neutrophil adhesion. Cells were then treated with 100 μl of the proteins secreted by JIR325 (6.3 mg/ml). Phorbol 12-myristate 13-acetate (PMA) (100 nM) was added as a positive control, and untreated neutrophils were used as a negative control in each experiment. In some experiments, the neutrophils were exposed to proteins secreted by JIR325 pre-incubated for 1 h with the DNAse inhibitor CuSO_4_ (0.01 M). These cells were incubated at 37°C in a humid atmosphere with 7% CO_2_. After the incubation, the cells were fixed with 4% paraformaldehyde, washed three times with PBS for 5 min, and then permeabilized with 0.5% Triton X-100 for 1 min at r.t. After 3 washes with PBS, blocking was performed with Protein Block Serum- (Dako, Denmark) in a humid chamber for 30 min at 37°C. Neutrophils were then incubated with the anti-neutrophil NE rabbit polyclonal antibody (Cat. No. 138 481001; EMD Millipore), diluted 1:100 in Tris-NaCl blocking buffer (TNB) for 1 h in a humid chamber at 37°C. After 3-5 -min washes with PBS, the coverslips were incubated for 1 h in a humid chamber at 37°C with a goat anti-rabbit IgG-FITC conjugate (Cat. No. ab6717; Abcam, USA), diluted 1:50 in TNB buffer. Finally, the coverslips were washed 3 times with PBS for 5 min and mounted on glass slides, using Mounting Medium with DAPI-Aqueous Fluoroshield (Cat. No. ab104139; Abcam, USA). Three assays were performed with three replicate samples (~1x10^4^ cells were analyzed per replicate).

### Semi-automated quantification of NETs

Images were captured using a Lionheart TM LX Automated Microscope (BioTek Instruments, Vermont, USA). An automatic protocol was implemented in the CellProfiler program (https://cellprofiler.org/) to analyze the fluorescence microscopy images. This protocol included two main steps according to the fluorescence channels: identifying total neutrophils (blue channel, DAPI) and identifying NE from NETs (green channel, FITC). Briefly, two independent steps with a function to identify primary objects were implemented to identify whole cells (size from 10 to 40 pixels) and NETs (size from 50 to 2000 pixels). Subsequently, a filtering step was performed to preserve only the cells that were releasing NETs or NETotic neutrophils using the mask functions in the program. The protocol was standardized with 10 sets of images and automatically applied to all images, including a quantification step. The performance was verified manually with the images generated from the analysis in all cases.

### Scanning Electron Microscopy (SEM) analysis

Neutrophils were incubated with the secreted proteins only or with these proteins plus 4x10^5^ *C. perfringens* for 60 min on poly-L-lysine pre-coated coverslips at 37°C and 5% CO_2_ atmosphere. Cells were fixed in 2% glutaraldehyde, post-fixed in 2% osmium tetroxide, and washed with phosphate buffer. Samples were then dehydrated with a graded ethanol series, stove-dried at 40°C for 48 h and sputtered with gold. All samples were visualized and analyzed with a JEOL Microscope model JSM6390LV.

### Nuclease activity assay

To measure nuclease activity, secreted proteins of *C. perfringens* were incubated with different concentrations of calf thymus DNA (1 µg, 0.5 µg, 0.250 µg, 0.125 µg, and 0.0625 µg) and 10X DNAse buffer (100 mM Tris-HCL, 25 mM MgCl_2_ and 1 mM CaCl_2_, pH 7.5) for 60 min at 37°C. Moreover, an experiment was carried out involving the incubation of supernatant with CuSO_4_ (0.01 M). The reaction was stopped by adding EDTA (50 mM) at 65°C for 10 min. Sterile RCM was used as a negative control. To visualize DNA degradation, 25 µl of the sample was mixed with 5 µl of gelRed (1:100 with loading buffer), and 20 µl of each sample was run on a 1.5% agarose gel.

### NETs bactericidal activity assay

*C. perfringens* culture at OD 600 = 0.6 was incubated with freshly isolated human neutrophils (4x10^5^ cells) exposed to *C. perfringens* secreted proteins or sterile RCM (negative control) for 30, 60, and 120 min at 37°C, 5% CO_2_ atmosphere under aerobic conditions. Serial dilutions of each treatment were performed after cell resuspension, and a volume of 100 µl per dilution was pipetted onto each RCM agar plate. Plates were incubated in an anaerobic atmosphere for 48-72 h, and the CFU was counted. Three assays were performed with three replicated samples.

### NETosis type determination

Neutrophils were treated with recombinant CpPLC (25.12 ng/ml) for 30 min, and cell viability was measured using the cell-permeable stain 4′,6-diamidino-2-phenylindole (DAPI) and the cell impermeable stain propidium iodide (PI). After incubation, cells were analyzed by immunofluorescence using a Lionheart automated microscope. Three assays were performed with three replicate samples (~1x10^4^ cells were analyzed per replicate).

### Recombinant CpPLC and polyclonal antibodies purification

The CpPLC gene from strain 8-6 was expressed in *E. coli*, and the recombinant toxin was purified as described (Alape-Girón et al., 2000). Briefly, the culture supernatant was loaded onto an FPLC anion-exchange Mono Q column equilibrated with 25 mM Bis-Tris-HCl, pH 6.8. The elution was performed with a gradient of NaCl (0 to 1 M) in Bis-Tris-HCl, pH 6.8. PLC active fractions were concentrated on a Macrosep system, loaded onto an FPLC gel filtration Superdex column, pooled, and ultrafiltered. The protein concentration in the final preparation was 1.25 mg/ml, and the purity of the recombinant protein was verified by SDS-PAGE and reverse-phase HPLC. The amount of endotoxin present in the recombinant CpPLC preparation was determined using the LAL assay, and it was found to be lower than 0.1 mg/ml. Polyclonal anti-CpPLC (38.6 mg/ml) or anti-bovine serum albumin (BSA) (6.2 mg/ml) antibodies were generated in rabbits, purified by caprylic acid precipitation, and concentrated by ultrafiltration.

### ROS detection assay

ROS generation in neutrophils was determined by incubating the cells with recombinant CpPLC and the ROS-sensitive probe dichlorodihydrofluorescein diacetate (DCFDA) (5 mM) for 30 min. After 3 washes with PBS, the percentage of fluorescent cells was determined using a Lionheart automated microscope. Three assays were performed with three replicate samples (~1x10^4^ cells were analyzed per replicate).

### Inhibition of CpPLC NETs and ROS induction

The capacity of the purified antibodies against CpPLC to neutralize the NETosis-inducing effect of the *C. perfringens* secreted proteins or recombinant CpPLC was determined by preincubation of the secreted proteins or the recombinant protein with serial 2-fold dilutions of the antibodies (initial concentration 1 mg/ml) for 1 h at 37°C. Several pharmacological inhibitors were used to test the dependence of CpPLC NETs or ROS induction on different mediators. The substances and concentrations used were as follows: the glutathione precursor N-acetyl-L-cysteine (NAC) (1-6 mg/ml); the free radical scavengers 4,5-dihydroxy-1,3-benzene disulfonic acid (tiron) (1-4 mg/ml), and pyrocatechol (50-200 µM); the mitochondrial-derived ROS scavenger MitoTEMPO (100-400 µM); the mitochondrial uncoupler 2,4-dinitrophenol (DNP) (200-800 µM); the NOX inhibitors diphenyleneiodonium chloride (DPI) (25-100 µM) and acetovanillone (0.1-0.4 mg/ml); the xanthine oxidase (XO) inhibitor allopurinol (4-16 mM); the inhibitors of arachidonic acid metabolism arachidonyl trifluoromethyl ketone (AACOCF_3_) (2.5-10 µM), and 5,8,11,14-eicosatetraenoic acid (ETYA) (12.5-50 µM): the noncompetitive antagonist of IP_3_ receptors 2-aminoethoxydiphenyl borate (2-APB) (50-400 µM); the scavenger of MPO-derived HClO luminol (50-200 µM); the PKC inhibitors bisindolylmaleimide I (GF109203X) (5-20 µM), safingol (7.5-30 µM) and hispidin (0.5-2 µM); the inhibitor of the MEK/ERK pathway U0126 (1.5-6 µg/ml). These drugs were not cytotoxic at the concentrations used because viability was greater than 95% in cells exposed only to the drugs compared to untreated cells.

A 96-well plate was coated with 0.001% poly-L-lysine for 1 h. Freshly isolated human neutrophils were seeded at a concentration of 7x10^4^ per well and incubated for 1 h with each inhibitor at 37°C and 5% CO_2_ atmosphere. Then, cells were exposed to recombinant CpPLC for 1 h as previously described and fixed with 4% paraformaldehyde. Cells were permeabilized with 0.5% Triton X-100 for 1 min at r.t. After 1-2 min washes with PBS, cells were stained with Höechst 33342 (31.25 µg/ml) for 10 min and washed twice with PBS for 1 min each. Finally, NETotic neutrophil quantification was done using the automatic protocol implemented in the CellProfiler program. Identification of total neutrophils (size from 10 to 40 pixels) and NETs (size from 50 to 2000 pixels) was differentiated with the size delimited for each object, followed by manual verification. Three assays were performed with three replicate samples (~1x10^4^ cells were analyzed per replicate).

### Statistical analysis

Results are expressed as mean +/- standard error of the mean (SEM) of at least three independent experiments. Statistical analysis was performed using one-way ANOVA followed by Tukey’s multiple comparison post-test. P values <0.01 and <0.05 were considered statistically significant.

## Results

### Proteomic analysis of *C. perfringens* secreted proteins

The concentrated supernatant proteins of *C. perfringens* toxinotype A strain JIR325 were separated by SDS-PAGE (**Supplementary Figure 1**). Each of the 17 bands was cut from the gel and analyzed by tandem mass spectrometry for peptide sequencing. A total of 14915 spectra corresponding to 4159 peptide sequences (the data are accessible at a public repository https://hdl.handle.net/10669/89483) were matched against the *C. perfringens* protein sequences in the UniProt database. The results allowed us to identify 39 proteins from the parental strain 13 (**Table 1**). The conserved domains of the secretome proteins were located by bioinformatic analysis using the CDD/SPARCLE database at NCBI (**Table 1**) (Lu et al., 2020). Furthermore, their biological function and subcellular localization were predicted with Gene Ontology at UniProtKB (**Table 1**) (Bateman et al., 2021). Thus *C. perfringens* secreted proteins were categorized as cytosolic, transmembrane, or extracellular.

**Table 1:** Proteomic analysis of the *Clostridium perfringens* toxinotype A secretome.

Avg. Mass(Da)	Accession (Uniprot)	Peptides (#)	Coverage (%)	-10lgP	Description	Gen/Locus (Genbank)	Domains (Accession)
189 894	Q8XKW1	77	39	353.05	Peptidase M60 domain- containing protein	CPE1281	F5/8 type C domain (pfam00754, 2 copies); Peptidase M60 (cl24257); DUF5011, (cl03620, 2 copies); Carbohydrate-binding (NPCBM; smart00776, 2 copies)
152 411	Q93M90	21	14	239.47	Probable collagen adhesin	*can* (plasmid encoded)	Prealbumin-like fold (pfam17802, 2 copies); Thioester domain (pfam08341)
133 260	Q8XJ10	34	25	300.07	2’,3’-cyclic nucleotide 2’- phosphodiesterase	*cpdA*; CPE1951	Multifunctional 2’,3’-cyclic-nucleotide 2’- phosphodiesterase/3’-nucleotidase/5’-nucleotidase (cl35825)
125 936	P43153	37	60	376.18	Collagenase colA	*colA*; CPE0173	Peptidase family M9 (pfam08453); Collagenase G (cl39822); PKD domain (pfam18911)
125 992	Q8XNP3	17	17	249.12	Endo-beta-N- acetylglucosaminidase	CPE0289	Endo-beta-N-acetylglucosaminidase D (cl44115): F5/8 type C domain (pfam00754); PKD repeat (COG3291); Fibronectin type 3 domain (cd00063); LPXTG-motif cell wall anchor domain (TIGR01167);
123 045	Q8XIL2	19	11	231.21	DUF1533 domain-containing protein	CPE2107	DUF1533 (cl06539)
122 462	Q8XL11	23	23	277.28	N-acetylglucosaminidase	CPE1231	Bacterial SH3 domain (pfam08239, 3 copies);YgiM (cl34551;4 copies); Beta-N-acetylglucosaminidase (cl27490)
96 690	Q8XNF8	30	34	277.50	Endo-beta-galactosidase C	CPE0375	F5/8 type C (2 copies; pfam00754); GH16_laminarinase_like (cd08023)
92 203	Q8XMR4	15	18	203.05	LYZ2 domain-containing protein	CPE0624	Beta-N-acetylglucosaminidase (cl27490); Pneumococcal surface protein PspC, choline-binding form (cl41462)
90 512	Q8XNK4	16	20	219.90	NPCBM domain-containing protein	CPE0329	NPCBM carbohydrate binding module (smart00776); Glycosyl hydrolase family 98M (cl07061)
81 511	Q8XIF9	6	7	151.86	Bifunctional metallophosphatase/5’- nucleotidase	*cpdC*; CPE2162	Multifunctional 2’,3’-cyclic-nucleotide 2’- phosphodiesterase/3’-nucleotidase/5’-nucleotidase (cl35825); LPXTG-motif cell wall anchor (TIGR01167)
77 314	Q8XMG4	43	43	343.87	Exo-alpha-sialidase	*nanI*; CPE0725	Non viral sialidase (cd15482)
66 482	P26823	20	30	234.94	Chaperone protein DnaK	*dnaK*; CPE0248	molecular chaperone DnaK (PRK00290)
63 368	Q8XMR3	18	28	254.26	Cell wall binding repeat- containing protein/zinc carboxypeptidase	CPE0625	Glucan-binding superfamily COG5263 (cl34963);M14 family of metallocarboxypeptidases (cl11393); pneumococcal surface protein PspC, choline-binding form (cl41462)
59 275	Q8XM44	26	29	328.14	Alpha-clostripain	*cloSI*; CPE0846	Cysteine protease Clostripain (TIGR02806)
58 688	Q8XKP0	8	14	192.07	SH3 domain/NlpC/P60 family protein	CPE1354	Bacterial SH3 domain (4 copies; pfam08239);Cell wall- associated hydrolase, NlpC family (COG0791)
57367	P26821	7	25	158.70	60 kDa chaperonin	*groEL*; CPE2289	chaperonin GroEL (PRK00013)
55 830	P0C2E9	31	49	336.87	Thiol-activated cytolysin (Perfingolysin)	*pfoA*; CPE0163	Thiol-activated cytolysin (pfam01289);Thiol-activated cytolysin beta 2lucosa2 domain (pfam17440)
54 346	Q8XIN7	12	22	198.17	DUF3502 domain-containing protein	CPE2078	Type 2 periplasmic binding fold (cl21456); DUF3502 (pfam12010)
54 258	Q8XLL1	14	24	219.30	DUF3502 domain-containing protein	CPE1030	Type 2 periplasmic binding fold (cl21456); DUF3502 (pfam12010)
53 324	Q8XMR2	5	13	154.88	YkuD domain-containing protein	CPE0626	Pneumococcal surface protein PspC, choline-binding form (cl41462); L,D-peptidoglycan transpeptidase YkuD (cl43946)
51 940	Q8XLN5	10	18	181.49	M18 family aminopeptidase	CPE1006	Zinc aminopeptidase 1 (PRK02256)
49 771	Q8XI54	9	16	188.89	Glucose-6-phosphate isomerase	*pgi*; CPE2267	Glucose-6-phosphate isomerase (PRK14097)
49 471	Q8XNH1	5	12	170.99	Pyruvate kinase	*pykA*; CPE2149	pyruvate kinase (cl35470)
47 721	Q8XHF5	10	19	154.57	Probable X-pro aminopeptidase	CPE2530	Aminopeptidase P (smart01011); Prolidase (cd01087)
47 217	P0C2E1	7	15	166.89	Arginine deiminase	*arcA*; CPE0168	arginine deiminase (PRK01388)
46 949	Q8XKU4	9	17	153.84	Enolase	eno; CPE1249	Enolase (PRK00077)
46 186	Q8XJF2	9	17	154.88	Pyrimidine-nucleoside phosphorylase	*deoA*; CPE1807	pyrimidine-nucleoside phosphorylase (cl42907)
45 530	P0C216	30	23	296.23	Phospholipase C (Alpha toxin)	CPE0036	Zinc dependent phospholipase C (smart00770); PLAT (cd00113)
43 980	Q8XHY1	18	33	242.22	probable maltose ABC transporter	CPE2343	periplasmic-binding component of ABC transport systems specific for maltose (cd13586)
42 555	Q8XNZ9	7	16	159.70	Cystathionine beta-synthase	*metB*; CPE0176	Aspartate aminotransferase I (cl18945)
38 741	Q8XNA0	10	27	175.08	Probable ion-uptake ABC transporter	CPE0438	Substrate binding domain of ferric iron-binding protein (cd13542)
37 153	P0C2E4	18	35	163.83	Ornithine carbamoyltransferase	*arcB*; CPE0169	ornithine carbamoyltransferase (PRK04284)
38 422	Q8XHK0	6	17	157.38	Foldase protein PrsA	*prsA*; CPE2483	peptidylprolyl isomerase (PRK00059)
36 700	Q8XKB3	6	16	150.76	ABC transporter substrate- binding protein	CPE1487	ABC-type nitrate/sulfonate/bicarbonate transport system (cl43248)
36 632	Q8XIW4	9	24	205.19	NAGPA domain-containing protein	CPE1999	Phosphodiester glycosidase (NAGPA; cl42026)
36 629	Q8XK52	6	22	171.18	Probable endo-1,4-beta- xylanase	CPE1551	NodB homology of S.mutans polysaccharide deacetylase PgdA (cd10944)
34 503	Q8XP50	9	22	184.08	N-acetylmuramoyl-L-alanine amidase domain-containing protein	CPE0115	N-acetylmuramoyl-L-alanine amidase (pfam01520)
29 991	Q8XN18	20	6	175.46	SGNH_hydro domain- containing protein	CPE0520	SGNH_hydrolase (cd00229)

The identified proteins include two major exotoxins: the CpPLC (Uniprot Accession number P0C216.1) and the thiol-dependent cytolysin, perfringolysin O (P0C2E9). Furthermore, *C. perfringens* secretome also contains eight secreted hydrolases (Q8XKW1, Q8XJ10, P43153, Q8XL11, Q8XNF8, Q8XMG4, Q8XM44, Q8XKP0), twelve cytosolic metabolic enzymes (Q8XNK4, Q8XLN5, Q8XI54, Q8XNH1, Q8XHF5, P0C2E1, Q8XKU4, Q8XJF2, Q8XNZ9, P0C2E4, Q8XK52; Q8XP50), two chaperones (P26823 and P26821), one folding enzyme (Q8XHK0), three membrane transporters (Q8XHY1, Q8XNA0, Q8XKB3), one adhesion membrane protein (Q93M90), two membrane enzymes (Q8XIW4 and Q8XN18), one secreted (Q8XIL2) and two periplasmic proteins (Q8XIN7 and Q8XLL1) of unknown functions, three cell wall-bound enzymes (Q8XMR4, Q8XMR3, Q8XMR2), and two cell wall-anchored enzymes (Q8XNP3 and Q8XIF9).

The two cell wall-anchored enzymes are a 126 kDa endo-beta-N-acetylglucosaminidase (Q8XNP3) and an 81.5 kDa bifunctional metallophosphatase/5’-nucleotidase (Q8XIF9). Amino acid sequence alignment analysis (**Supplementary Figure 2**) revealed that the *C. perfringens* Q8XIF9 bifunctional enzyme shows significant amino acid sequence identity to the virulence-associated *S. pyogenes* S5nA (Uniprot accession number A0A5S4TI37) (18.7%), the A0A3Q8B6L4 *Streptococcus suis* AdsA (19.4%) and the A3CKJ7 Nt5e *Streptococcus sanguinis* (24.0%) cell anchored 5´nucleotidases (Fan et al., 2012; Zheng et al., 2015; Dai et al., 2018). The alignment shows that the conserved positions are grouped into various motifs distributed throughout the polypeptide chains and revealed that several of the strictly conserved residues are glycine (7 glycine residues are invariant), typical findings in homologous proteins with similar three-dimensional structure.

### *C. perfringens* secreted proteins induce NETs formation by human neutrophils

**Figure 1:**
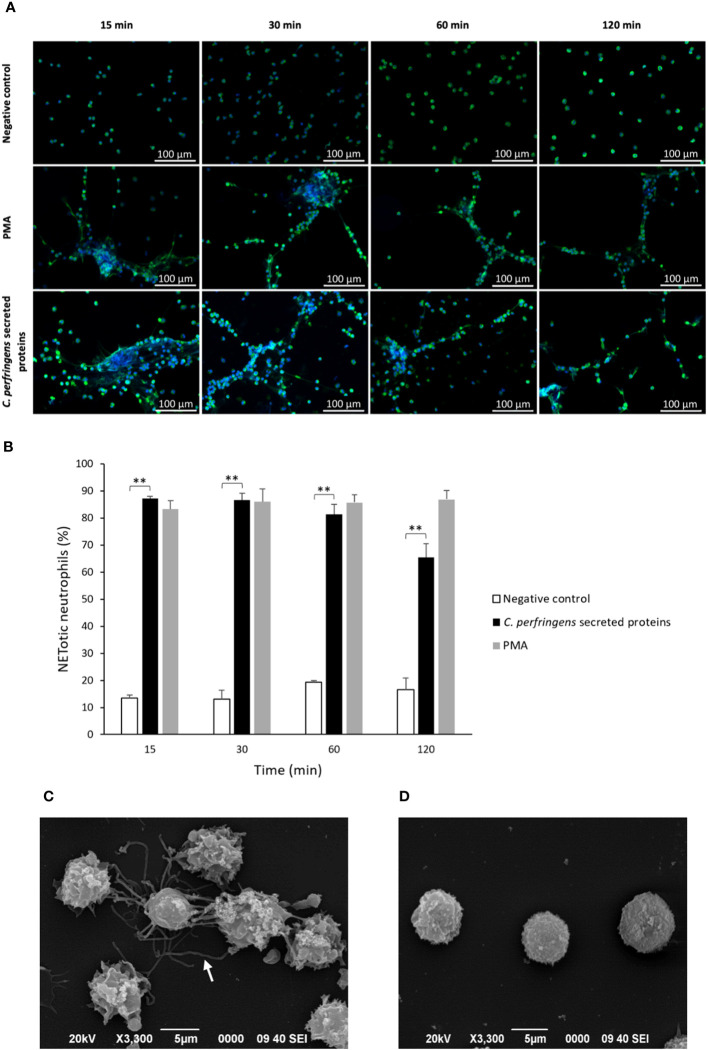
*C. perfringens* secreted proteins induce NETs formation by human neutrophils *in vitro*. **(A)** Determination of NETs formation by immunofluorescence (green-elastase and blue-DNA) at 15, 30, 60, and 120 min using *C. perfringens* secreted proteins as a stimulus. Ultrafiltered culture medium and PMA were used as negative and positive controls, respectively. **(B)** Quantitative analysis of the percentage of NETotic human neutrophils when exposed to *C. perfringens* secreted proteins. Results show means **±** SEM. Treatments with statistically significant differences compared to the control are indicated by ** (*P* < 0.01). **(C)** Scanning electron micrograph of NETs induced by *C. perfringens* secreted proteins in human neutrophils *in vitro.* The white arrow indicates NETs-like structures. **(D)** Negative control.

Neutrophils were exposed to *C. perfringens* secreted proteins at different times, and NETs induction was studied using immunofluorescence or SEM. Immunofluorescence microscopy analysis showed decondensed chromatin fibers and NE extracellular release by stimulated neutrophils, characteristic of NETs induction (**Figure 1A**). NETs are induced in 85% of the total amount of neutrophils compared to 15% in unstimulated cells after 15 min of exposure to proteins secreted by *C. perfringens* (**Figure 1B**). However, NETs induced by *C. perfringens* secreted proteins diminished on time, with 20% NETs reduction observed from 60 min to 120 min, suggesting that NETs are degraded (**Figure 1B**). SEM analysis showed characteristic NETs like structures (**Figure 1C**), in contrast to CpPLC untreated neutrophils (**Figure 1D**).

### NETs kill *C. perfringens*

**Figure 2:**
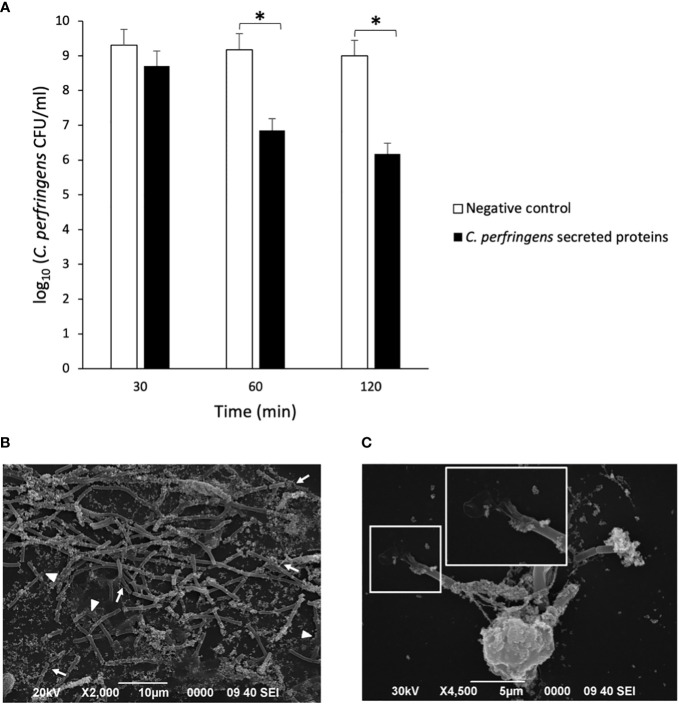
NETs kill *C. perfringens*. **(A)** Survival of *C. perfringens* (~10^6^ CFU/ml) incubated with human neutrophils exposed or not to *C. perfringens* secreted proteins for 30, 60, and 120 min. Results show means **±** SEM. Significant differences in the samples compared to the control are indicated by * (*P* < 0.05). **(B, C)** Scanning electron micrograph of *C. perfringens* being captured by NETs released from a human neutrophil *in vitro* after the exposure to *C. perfringens* secreted proteins. The white arrows indicate NETs, and the white arrowheads point to partially lysed bacteria; the inset shows a direct membrane lesion of the bacteria trapped in the NETs.

The survival of *C. perfringens* (~10^6^ CFU/ml) incubated with human neutrophils exposed or not to *C. perfringens* secreted proteins was measured at 30, 60, and 120 min (**Figure 2A**). The results showed a statistically significant decrease in bacterial survival when NETs are induced (**Figure 2A**), indicating a direct microbicidal effect of NETs on *C. perfringens.* Furthermore, electron microscopy analysis shows that the fibrous structure of NETs ensnares *C. perfringens* (**Figure 2B**) and reveals a direct membrane lesion of the bacteria trapped in the NETs (**Figure 2C**).

### *C. perfringens* scape from NETs

**Figure 3:**
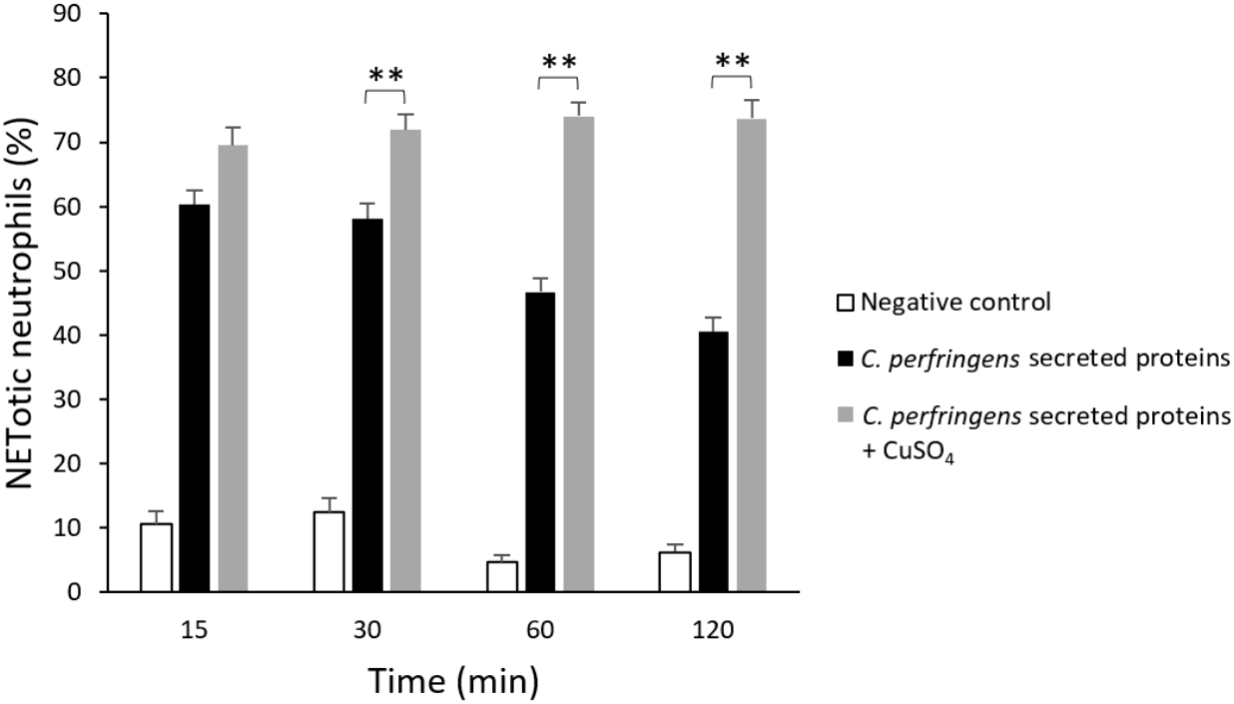
DNase present in *C. perfringens* secreted proteins degrades NETs and CuSO_4_ inhibits its activity. Analysis of the percentage of NETotic human neutrophils *in vitro* when exposed to the *C. perfringens* secretome for 15, 30, 60, and 120 min in the absence and the presence of the DNAse inhibitor CuSO_4_. Ultrafiltered culture medium was used as a negative control. Results show means **±** SEM. Significant differences between samples are indicated by ** (*P* < 0.01).

Previous studies indicated that some bacteria, such as *S. aureus* and Group A Streptococcus, produce DNAses to escape from NETs entrapment (Liao et al., 2022). Since there is a reduction of NETs over time when neutrophils are treated with *C. perfringens* secreted proteins (**Figure 1B**), the percentage of NETotic human neutrophils following exposure to the secreted proteins and the DNAse inhibitor CuSO_4_ for 15, 30, 60, and 120 min was measured (**Figure 3**). The results show significant dose-dependent differences in NETs degradation in the presence of the DNAse inhibitor (**Figure 3**). Furthermore, electrophoresis analysis indicates that *C. perfringens* secreted proteins exhibit nuclease activity on calf thymus DNA (**Supplementary Figure 3**). The DNAse activity of the secreted proteins measured as the DNA degradation in gel electrophoresis is inhibited when samples are exposed to CuSO_4_ (**Supplementary Figure 4**).

### The Q8XKM6 DNAse is present in the *C. perfringens* secretome

The presence of DNAse activity in the culture supernatants of different *C. perfringens* strains has been shown previously (Porschen and Sonntag, 1974), and the current results demonstrate DNAse activity in the JIR325 *C. perfringens* strain secretome (**Supplementary Figures 3**, **4**). A directed search was performed to identify the protein responsible for this activity, matching the peptide spectra of the *C. perfringens* secreted proteins against the sequences of the clostridial DNAses contained in UniProt (**Supplementary Table 1**). A total of 157 spectra corresponding to 52 peptide sequences (**Supplementary Table 2**) matched 26% of the sequence (-10lgP = 65.5) of a previously cloned and characterized cell wall-anchored 190 kDa LTD domain-containing DNAse (Okumura et al., 2005) (UniProt Accession number Q8XKM6) (**Supplementary Figure 5**). This strategy helped identify the *C. perfringens* DNAse in the secretome. Similarly, several streptococcal species produce cell wall-anchored DNAses, also found in culture supernatants (Hasegawa et al., 2010; Chang et al., 2011; de Buhr et al., 2014; Morita et al., 2014). Indeed, an amino acid sequence alignment (**Supplementary Figure 6**) revealed that the *C. perfringens* Q8XKM6 DNAse shows significant amino acid sequence identity to the virulence-associated *S. pyogenes* SpnA (Uniprot accession number A0A455ZLVU4) (19.3%), the Q6X5T7 *S. suis* ssnA (20.8%) and the A3CPM7 *S. sanguinis* (20.6%) cell anchored nucleases (Hasegawa et al., 2010; Chang et al., 2011; de Buhr et al., 2014; Morita et al., 2014).

### *C. perfringens* PLC induces NETs

**Figure 4:**
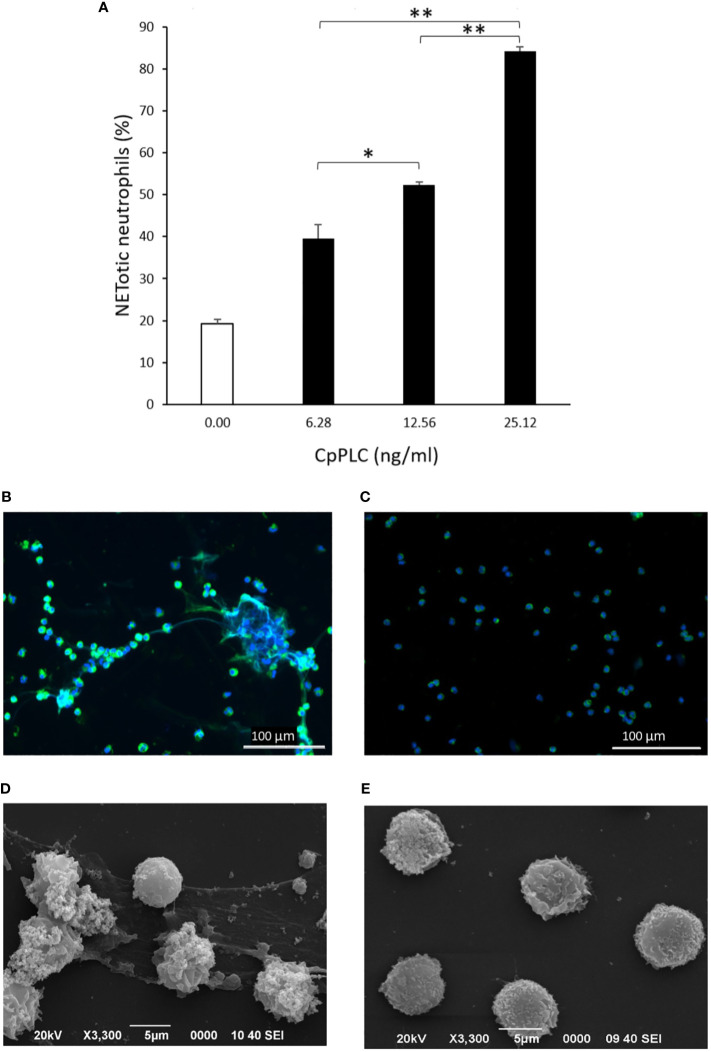
Recombinant CpPLC induces NETs formation by human neutrophils *in vitro*. **(A)** Quantitative immunofluorescence analysis of NETs induced by CpPLC after 60 min of exposure. Results show means **±** SEM of two independent experiments with three replicates each. Significant differences between samples are indicated by * (*P* < 0.05) and ** (*P* < 0.01). **(B)** CpPLC induces NETs formation after one hour, as shown by immunofluorescence (green-elastase and blue-DNA). **(C)** Unstimulated neutrophils (Negative control). **(D, E)** Electron microscopy micrographs of NETs after exposure of human neutrophils *in vitro* to recombinant CpPLC and negative control, respectively.

**Figure 5:**
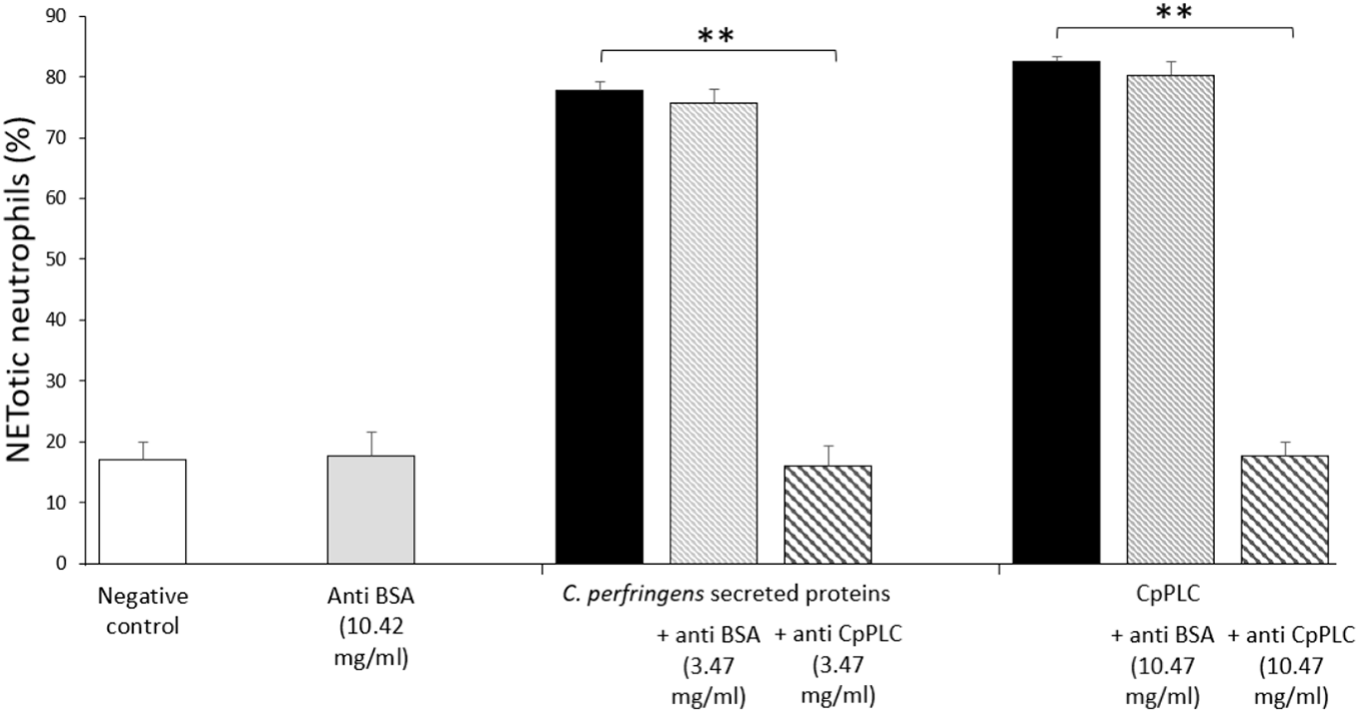
Antibodies against CpPLC inhibit NETosis induced by *C. perfringens* secreted proteins and recombinant CpPLC. Quantitative analysis of NETotic human neutrophils inhibition when exposed to *C. perfringens* secretome or CpPLC previously preincubated with anti-CpPLC or anti-bovine serum albumin (BSA) antibodies. Neutrophils unstimulated or exposed to anti-BSA antibodies were used as controls. Results show means **±** SEM. Significant differences between treatments are indicated by ** (*P* < 0.01).

Since CpPLC is the main toxin produced by *C. perfringens* toxinotype A, its possible role in inducing NETs by human neutrophils was assessed (**Figure 4A**). Immunofluorescence (**Figure 4B**) and SEM analysis (**Figure 4D**) of human neutrophils after 1 h exposure to recombinant CpPLC shows NETs formation, in contrast to untreated neutrophils (**Figures 4C, E**). The results showed that CpPLC induces NETs in human neutrophils in a dose-dependent manner (**Figure 4A**). Polyclonal antibodies specific to CpPLC completely neutralize NETosis caused by the *C. perfringens* secreted proteins and by recombinant CpPLC (**Figure 5**).

### CpPLC induces suicidal NETosis

**Figure 6:**
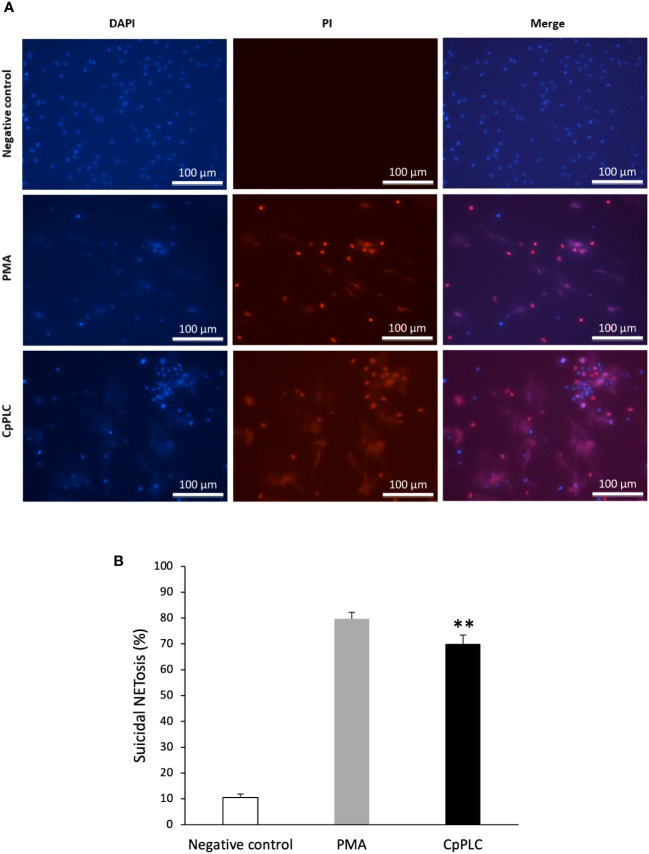
CpPLC induces suicidal NETosis. **(A)** Neutrophils viability was analyzed after CpPLC exposure with the cell-permeable stain DAPI (blue) and the cell-impermeable stain PI (red), as described in Materials and methods. Ultrafiltered culture medium and PMA were used as negative and positive controls, respectively. **(B)** Quantitative analysis of the percentage of neutrophils that underwent to suicidal NETosis when exposed to CpPLC. Results show means **±** SEM. Significant differences in the treatments compared to the control are indicated by ** (*P* < 0.01).

The type of NETosis induced by CpPLC in human neutrophils was determined by measuring cell survival. Vital neutrophils were identified with DAPI, and death neutrophils with PI. A kinetic experiment showed that neutrophils exposed to CpPLC, which become NETotic, immediately incorporate the cell impermeable stain PI (**Figure 6A**), demonstrating that this toxin significantly induces suicidal NETosis compared with the control (**Figure 6B**).

### CpPLC induces ROS-dependent NETosis stimulating storage operated calcium entry

**Figure 7:**
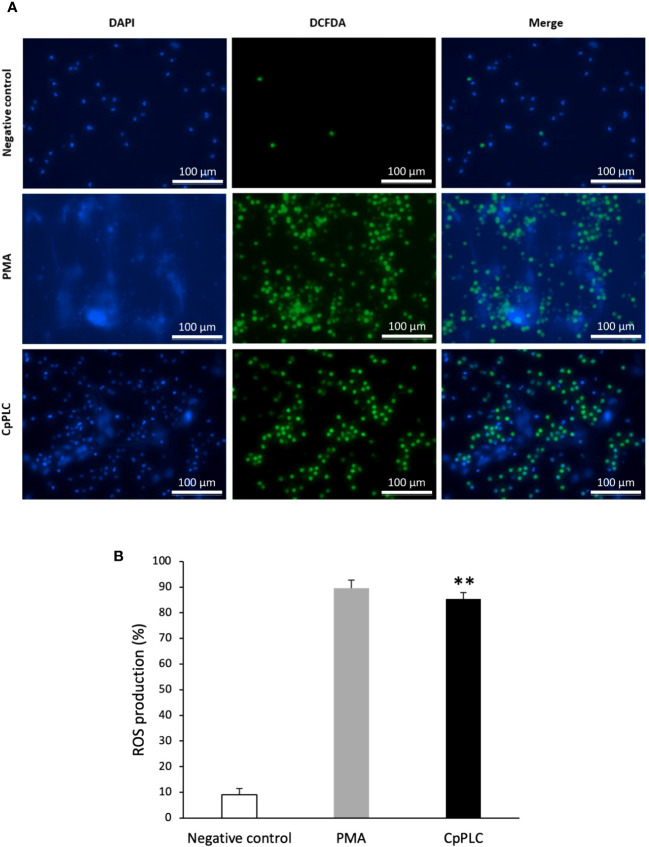
ROS are induced in human neutrophils exposed to CpPLC. **(A)** ROS production in human neutrophils exposed to CpPLC was evaluated by fluorescence microscopy using the cell-permeant dye DCFDA (green) and the cell-permeable stain DAPI (blue). Ultrafiltered culture medium was used as negative control and PMA as positive control. **(B)** Quantitative analysis of ROS production when exposed human neutrophils to CpPLC. Results show means **±** SEM. Significant differences in the treatments compared to the control are indicated by ** (*P* < 0.01).

ROS production during NETosis induced by CpPLC was evaluated in human neutrophils using the cell-permeable and oxidant-sensitive probe DCFDA after toxin exposure. When DCFDA is taken by the cells and the ROS oxidase it, the highly fluorescent 2’,7’–dichlorofluorescein (DFC) is produced. The results show that neutrophils became fluorescent and NETotic after exposure to CpPLC (**Figure 7A, B**), indicating that ROS are involved in the activated NETosis pathway. The critical role of ROS in this pathway was established by the dose dependent NETosis inhibition accomplished by the treatment with different scavengers (**Table 2**). The treatment with NAC and tiron, pyrocatechol, and luminol inhibits NETs formation in a dose-dependent manner (**Supplementary Figures 7A-D**), even reaching a complete inhibition with NAC at 6 mg/ml (**Supplementary Figure 7A**).

**Table 2:** Effects of antioxidants and signal transduction pathways inhibitors on NETs formation and ROS production induced by CpPLC.

Substance (concentration)	Mode of action/Target	NET formation inhibition (%)	ROS inhibition (%)
Antioxidants			
N-acetyl-L-cysteine (6 mg/mL)	Glutathione precursor	100 ± 0.58	100 ± 4.13
Tirón (4 mg/mL)	Free radical scavenger	63 ± 1.77	33 ± 2.59
Pyrocatechol (100 μM)	Free radical scavenger	53 ± 2.70	47 ± 3.89
Luminol (200 μM)	HClO scavenger	68 ± 1.83	52 ± 3.17
MitoTEMPO (400 μM)	Mitochondrial ROS scavenger	0	N/A
2,4-dinitrofenol (2,4-DNP) (800 μM)	Mitochondrial uncopler	0	N/A
Diphenylene iodonium (DPI) (100 μM)	NADPH oxidase inhibitor	0	N/A
Acetovanillona (0.4 mg/mL)	NADPH oxidase inhibitor	0	N/A
AACOCF3 (10 μM)	Arachidonic acid metabolism inhibitor	50 ± 3.26	37 ± 6.20
Eicosatetraynoic acid (12.5 μM)	Arachidonic acid metabolism inhibitor	46 ± 2.08	44 ± 4.08
Allopurinol (16 mM)	Xanthine oxidase (XO) inhibitor	93 ± 1.29	97 ± 0.94
Signal transduction pathways inhibitors			
Aminoethoxydiphenyl borate (400 μM)	ER Ca^2+^ pumps	94 ± 1.56	48 ± 5.62
U0126 (1.5 μg/ml)	MEK/ERK	55 ± 2.25	57 ± 3.46
GF109203X (20 μM)	PKC	93 ± 1.69	25 ± 2.57
Safingol (30 μM)	PKC	58 ± 3.03	38 ± 4.67
Hispidin (2 μM)	PKC	58 ± 2.74	14 ± 3.62

Data represent the average of three independent experiments with three replicates each.

Data are presented as mean ± SEM.N/A, Not assessed.

To assess the role of store-operated calcium entry in the NETosis pathway triggered by CpPLC, we evaluated the effect of preincubating the neutrophils with 2-APB before toxin exposure (**Supplementary Figure 7E**). The data clearly show that IP_3_ receptors antagonist effectively prevents NETs formation (**Supplementary Figure 7E**). Furthermore, inhibition of calcium entry with 2-APB partially inhibits ROS generation as it decreases the fluorescence intensity of DFC-labeled neutrophils (**Supplementary Figure 7E**).

Different inhibitors were used to clarify the ROS source and the signal transduction pathways required for NETs induction (**Table 2**). Results show that the mitochondrial ROS scavenger MitoTEMPO or the mitochondrial uncoupler DNP do not affect NETs formation triggered by CpPLC. Furthermore, NETosis induced by this toxin is not affected by the NOX inhibitors DPI or acetovanillone (**Table 2**). In contrast, NETs formation induced by CpPLC partially decreases in the presence of the inhibitors of arachidonic acid metabolism AACOCF_3_ and ETYA, and remarkably, it is almost completely inhibited by the XO inhibitor allopurinol (**Table 2**; **Supplementary Figures 7F-H**).

Furthermore, the roles of the PKC and MEK-ERK pathways in the CpPLC induced NETosis were assessed using inhibitors of those kinases (**Table 2**). Results show that NETs induction by CpPLC is partially inhibited by the PKC inhibitors GF109203X, safingol, hispidin, and the MEK inhibitor U0126 (**Table 2**; **Supplementary Figures 7I-L**).

## Discussion

The proteomic analysis of *C. perfringens* secreted proteins at the stationary phase revealed 40 proteins. Some identified proteins are known to play a role in virulence, helping bacteria in host colonization, immune suppression, or nutrient acquisition for bacterial growth, and include several exotoxins and secreted hydrolases (Rood et al., 2018; Mehdizadeh Gohari et al., 2021).

The secreted proteins, which do not contain any signal peptide, were classified as cytosolic enzymes, such as pyruvate kinase and enolase, or housekeeping proteins, like the GroEL and dnaK chaperones. Besides their metabolic role, some of these enzymes are moonlighting proteins and have unexpected extracellular functions, such as promoting adhesion or immune evasion (Ebner and Götz, 2019). The GroEL and dnaK chaperones facilitate protein folding, cell wall organization, and stress response encountered in host tissues during infection (Ebner and Götz, 2019).

Three cell wall-anchored proteins were also identified: the Q8XNP3 endo-beta-N-acetylglucosaminidase, the Q8XIF9 5’-nucleotidase, and the Q8XKM6 DNAse. These cell wall-anchored proteins could be released into the extracellular milieu during bacterial growth, either as by-products of cell wall turnover or by specific hydrolases (Fischetti, 2019).

Besides the cell wall-anchored Q8XIF9 5´-nucleotidase, the *C. perfringens* secretome contains a 133 kDA Q8XJ10 5´-nucleotidase (**Table 1**). *C. perfringens* 13 strain genome also encodes a 130 kDa 5’-nucleotidase containing a cell wall-anchored LPXTG-motif (Uniprot accession number Q8XJ11). These three *C. perfringens* 5´nucleotidases share 36.7-66.0% sequence identity. They are homologous to enzymes relevant for pathogenicity in different streptococcal species (Soh et al., 2020). 5´-nucleotidases catalyze the hydrolytic dephosphorylation of nucleotides and deoxynucleotides to their respective nucleosides and deoxynucleosides (Zakataeva, 2021). Cell wall-anchored or secreted bacterial 5´-nucleotidases could be virulence factors that disturb the host inflammatory response (Zakataeva, 2021). During infection-associated tissue damage, an increase in nucleotide adenosine triphosphate (ATP) from the extracellular milieu induces the release of several inflammatory mediators (Di Virgilio et al., 2020). The extracellular ATP dephosphorylation by 5´-nucleotidases increases adenosine concentration, constituting a negative feedback signal that suppresses the immune response (Di Virgilio et al., 2020). Adenosine accumulation helps the pathogen subvert the host’s immune response and favors its survival (Zakataeva, 2021). Further studies are warranted to evaluate the potential contribution of the extracellular 5´-nucleotidases to C. *perfringens* virulence.

In this study, we report that *C. perfringens* secreted proteins at the stationary phase induce NETs formation by human neutrophils, as shown by immunofluorescence and SEM analysis (**Figure 1**). The bacterial survival measured as CFU shows that the viability of *C. perfringens* diminishes when NETs are induced (**Figure 2A**), indicating a direct microbicidal effect of NETs on *C. perfringens*, supported by SEM analysis (**Figures 2B, C**).

A relevant finding was that NETs induced by *C. perfringens* secreted proteins diminished over time, with a 20% NETs reduction observed from 60 to 120 min (**Figure 1B**). This result suggest that the bacteria could produce a DNAse to evade NETs. Further studies revealed that the DNAse inhibitor CuSO_4_ prevents NETs degradation by these secreted proteins (**Figure 3**). In addition, electrophoresis DNA analysis shows that *C. perfringens* secreted proteins exhibit nuclease activity on calf thymus DNA, and CuSO_4_ inhibited that activity (**Supplementary Figure 4**). However, the initial proteomic analysis did not show any protein with nuclease activity in the *C. perfringens* secretome. Therefore, a more directed proteomic analysis searching for a DNAse revealed a previously characterized 190 kDa *C. perfringens* Q8XKM6 nuclease. An amino acid sequence alignment of this nuclease (**Supplementary Figure 6**) shows that it is homologous to other cell wall-anchored nucleases from *S. pyogenes*, *S. suis*, and *S. sanguinis* involved in survival and colonization fitness (Liao et al., 2022). Cell wall-anchored DNAse lacking strains of *S. pyogenes*, *S. suis*, and *S. sanguinis* show increased bacterial resistance to neutrophil elimination (Hasegawa et al., 2010; Chang et al., 2011; de Buhr et al., 2014; Morita et al., 2014). Furthermore, a cell wall-anchored SpnA DNAse knockout of *S. pyogenes* is less lethal than the parental strain in a mouse infection model, demonstrating a role in virulence (Morita et al., 2014). A synergic effect in pathogenicity between cell wall-anchored 5´-nucleotidases and DNAses expressed in Gram-positive cocci has been described (Soh et al., 2020). The 5´-nucleotidases dephosphorylate the dAMP released from the NETs DNA backbone by DNAses and produce deoxy-adenosine, which triggers macrophage apoptosis, thus resulting in a “double hit” strategy of immune evasion (Soh et al., 2020). Interestingly, in a murine model of gas gangrene, the expression of the Q8XKM6 DNAse as well as the Q8XL11 and the Q8XJ10 5´-nucleotidases occurs 1.5 h after intramuscular inoculation of 10^9^ C*. perfringens* vegetative cells (Low et al., 2018). Further studies are warranted to assess the potential synergism of Q8XKM6 DNAse and those 5´-nucleotidases during the pathogenesis of *C. perfringens* infections.

CpPLC, the first bacterial toxin found to be enzymatically active, is hemolytic, cytotoxic, induces platelet aggregation and myonecrosis, which altogether leads to lethality (Monturiol-Gross et al., 2021). The data conclusively demonstrated that this toxin is the NET inductor in *C. perfringens* secreted proteins. Recombinant CpPLC induces NETs formation by human neutrophils in a dose-dependent manner (**Figure 4A**). Furthermore, polyclonal antibodies against CpPLC completely neutralize NETosis triggered by the *C. perfringens* secreted proteins (**Figure 5**). A higher amount of the same antibodies preparation also neutralizes the NETosis induced by the recombinant CpPLC (**Figure 5**). Different concentrations of antibodies needed to neutralize the NETotic effect is probably related to lower CpPLC concentration in the *C. perfringens* secretome.

NETosis can proceed through diverse pathways depending on the triggering stimulus (Kenny et al., 2017). Those pathways differ in the receptors activated, the signaling transduction mechanisms, and intracellular mediators (Thiam et al., 2020; Singh et al., 2023). Calcium is an essential second messenger required for NETosis, and therefore, its cytosolic increase is a critical factor in all NETs-inducing pathways (Thiam et al., 2020; Singh et al., 2023). The calcium required for NETs formation could enter from the extracellular space or intracellular stores (Thiam et al., 2020; Singh et al., 2023). CpPLC induced NETosis requires store-operated calcium entry, as the noncompetitive antagonist of IP_3_ 2-APB almost completely prevents NETs formation (**Supplementary Figure 7E**).

ROS are also required as mediators in most NETosis activating signaling pathways, although, in some conditions, NETs can be induced independently of ROS (Stoiber et al., 2015). The pathway activated by CpPLC is ROS-dependent, as various scavengers prevent the NET formation caused by this toxin. NETosis induced by CpPLC does not require mitochondrial derived ROS since the mitochondrial ROS inhibitor MitoTEMPO or the mitochondrial uncoupler DNP does not inhibit it (**Table 2**). Furthermore, the NETosis pathway activated by CpPLC is not affected by the NOX inhibitors DPI and acetovanillone (**Table 2**). Therefore, CpPLC induces NETosis *via* activating a ROS-dependent pathway in which neither the mitochondria nor the NOX complex is the ROS source.

Since the XO inhibitor allopurinol blocks ROS production and almost entirely inhibits the NETosis induced by CpPLC (**Supplementary Figure 7H**), XO seems to be an important source of the ROS required for NETs formation triggered by this toxin. Similarly, it has been previously reported that allopurinol significantly prevents NETosis induced by *S. aureus* Panton-Valentine Leukocidin (Mazzoleni et al., 2021).

Calcium and ROS signaling are interconnected during NETosis, since calcium can increase ROS production, whereas ROS modulate the activity of various calcium transporters (Görlach et al., 2015; Thiam et al., 2020; Singh et al., 2023). Calcium seems to be contributing to ROS production in the NETosis pathway activated by CpPLC, as inhibition of calcium entry with 2-APB decreases the fluorescence of DFC-labeled neutrophils (**Supplementary Figure 7E**).

Suicidal NETosis induced by the PKC activator PMA and many other stimuli requires store-operated calcium entry, activation of the MEK-ERK pathway, and NOX and MPO derived ROS (Hakkim et al., 2011; Metzler et al., 2014; Björnsdottir et al., 2015; Damascena et al., 2022). Vital NETosis is NOX-independent and requires extracellular calcium entry mediated by SK3, activation of MEK/ERK, and mitochondrial ROS (Thiam et al., 2020; Singh et al., 2023). We have previously reported that CpPLC induces ROS production in a PKC- and MEK/ERK-dependent manner (Monturiol-Gross et al., 2012). The results of this work (**Table 2**) demonstrate that CpPLC induces suicidal NETosis through a pathway that involves PKC activation and the MEK/ERK pathway, requiring calcium entry as well as XO and MPO derived ROS.

The effects of CpPLC on skeletal muscle and its vasculature have been studied by intravital microscopy (Alape-Girón et al., 2000; Bryant et al., 2000a; Bryant et al., 2000b). CpPLC induces the formation of mobile intravascular aggregates that grow in number and size, which leads to progressive occlusion of small, medium, and large vessels (Alape-Girón et al., 2000; Bryant et al., 2000a; Bryant et al., 2000b), which enhances the ischemia and extends the anaerobic environment required for *C. perfringens* growth and spread within the infected tissue. Interestingly, the blood perfusion deficit induced by CpPLC is entirely preventable by pretreatment of the animals with anti-neutrophil antibodies (Bryant et al., 2000b).

NETs can stimulate many platelet signaling pathways that trigger platelet-driven coagulation (Wienkamp et al., 2022). Some NETs derived proteins, such as histones H3 and H4 and cathepsin G, directly activate platelets and trigger their aggregation (Wienkamp et al., 2022). Furthermore, the negatively charged surface provided by DNA within the NETs scaffold constitutes a procoagulant surface, further contributing to microthrombi formation (Wienkamp et al., 2022). The findings of this work raise the possibility that intravascular CpPLC induced NETs could play a role in the thrombotic events at the microvasculature of the *C. perfringens* infected tissues.

On the other hand, activated platelets may directly promote neutrophil NETs formation by an interaction mediated by the P-selectin and indirectly through mediators released during their activation, such as the thromboxane A2, the alarmin HMGB1, and the defensin hBD-1 (Wienkamp et al., 2022). Since CpPLC induces the formation of P-selectin positive activated platelet aggregates, it is also possible that CpPLC generates NETs indirectly through platelet activation.

The induction of NETs by CpPLC requires ROS, and previously, we demonstrated that antioxidants reduce myonecrosis and lethality in a murine model of gas gangrene (Monturiol-Gross et al., 2012). The results of this work warrant further studies to clarify whether antioxidants reduction of myotoxicity and lethality caused by *C. perfringens* induced gas gangrene is due to NETosis inhibition.

The muscle regeneration process after myonecrosis caused by *C. perfringens* in a murine gas gangrene model has been previously studied (Zúñiga-Pereira et al., 2019). Skeletal muscle frequently has a high regeneration capacity, and after the injury, neutrophils have a critical role as promoters of the inflammatory response required for tissue repair (Torres-Ruiz et al., 2023). However, a dysregulation of the immune response with an enhanced production and/or a deficient degradation of NETs after muscle injury could contribute to increased muscle damage and hamper tissue repair (Torres-Ruiz et al., 2023). After experimental gas gangrene, there is impaired muscle regenerative activity (Zúñiga-Pereira et al., 2019). Further studies are required to determine whether it is due to NETs excess in the infected tissue.

This work reveals an unknown effect of a bacterial phospholipase that could be relevant for understanding the pathogenesis of gas gangrene. The results suggest that *C. perfringens* has developed a combination of virulence factors that target NETs formation and degradation, thus enabling the bacterium to manipulate the innate immune response for its benefit and later escape to adjacent tissue. Other bacterial pathogens associated with soft tissue infections could use a similar virulence strategy during infection establishment and progression. Understanding the role of NETs in the thrombotic events that occur in gas gangrene holds the potential to develop novel therapeutic strategies to reduce mortality, improve muscle regeneration, and prevent deleterious outcomes for patients.

## Data availability statement

The datasets presented in this study can be found in online repositories. The names of the repository/repositories and accession number(s) can be found in the article/**Supplementary Material**.

## Ethics statement

The studies involving human samples were approved by Institutional Ethics Committee, University of Costa Rica. The studies were conducted in accordance with the local legislation and institutional requirements. The human samples used in this study were acquired from blood provided by healthy volunteers. Written informed consent for participation was not required from the participants in accordance with the national legislation and institutional requirements.

## Author contributions

LB-V: Writing – original draft, Writing – review & editing, Data curation, Formal Analysis, Investigation, Methodology, Visualization. RP: Investigation, Data curation, Formal Analysis, Methodology, Visualization, Writing – review & editing. JM-M: Formal Analysis, Methodology, Software, Visualization, Writing – review & editing. AA-G: Writing – original draft, Writing – review & editing, Data curation, Formal Analysis, Methodology, Resources, Visualization. MF-D: Writing – original draft, Writing – review & editing, Conceptualization, Data curation, Formal Analysis, Funding acquisition, Investigation, Methodology, Project administration, Resources, Supervision, Validation, Visualization.

## Supplementary material

Supplementary Figure 1 SDS-PAGE of *C. perfringens* secreted proteins at the stationary phase. *C. perfringens* secreted proteins were separated by SDS-PAGE and stained with Coomassie Brilliant Blue. The bands were cut out for in gel digestion and subsequent peptide analysis by mass spectrometry.

Supplementary Figure 2 Sequence alignment of the *C. perfringens* C8XIF9 bifunctional metallophosphatase/5’-nucleotidase with streptococcal 5´-nucleotidases. Accession codes in the Uniprot database are given on the left. Residues conserved in all sequences are indicated with an asterisk, and positions strictly conserved are indicated with two dots.

Supplementary Figure 3 C*. perfringens* secreted proteins degrade DNA. Agarose gel electrophoresis shows the result of calf thymus DNA degradation by *C. perfringens* secreted proteins. Lanes 1, 3, 5, 7, and 9: calf thymus DNA control 1 µg, 0.5 µg, 0.250 µg, 0.125 µg and 0.0625 µg respectively. Lanes 2, 4, 6, 8, and 10: calf thymus DNA 1 µg, 0.5 µg, 0.250 µg, 0.125 µg, and 0.0625 µg, respectively exposed to the proteins secreted by *C. perfringens* to the supernatants. M: Thermo Fisher molecular weight marker Mass Ruler High Range DNA Ladder (#SM0393).

Supplementary Figure 4 CuSO_4_ inhibits DNA degradation by *C. perfringens* secreted proteins in a dose-dependent manner. Agarose gel electrophoresis shows the result of calf thymus DNA degradation by *C. perfringens* secreted proteins. Lanes 1, 4, and 7: calf thymus DNA control 0.250 µg, 0.125 µg, and 0.0625 µg, respectively. Lanes 2, 5, and 8: calf thymus DNA 0.250 µg, 0.125 µg, and 0.0625 µg, respectively, exposed to *C. perfringens* secreted proteins. Lanes 3, 6, and 9: the same previous set of samples and exposed to CuSO_4_. M: Thermo Fisher molecular weight marker MassRuler High Range DNA Ladder (#SM0393).

Supplementary Figure 5 Amino acid sequence of the DNAse present in the *C. perfringens* secretome. The amino acid sequence of the cell-wall anchored DNAse (Uniprot Accession number Q8XKM6) is shown. The shadowed segments correspond to the peptides identified by tandem mass spectrometry after in gel digestion of *C. perfringens* secreted proteins.

Supplementary Figure 6 Sequence alignment of the *C. perfringens* Q8XKM6 DNAse with streptococcal nucleases. Accession codes in the Uniprot database are given on the left. Residues conserved in all sequences are indicated with an asterisk, and positions strictly conserved are indicated with two dots.

Supplementary Figure 7 Antioxidants and signal transduction pathways inhibitors blocked ROS production and NETs induction by CpPLC. Dose-dependent inhibition of ROS production and NETs formation by **(A)** NAC, **(B)** Tiron, **(C)** Pyrocatechol, **(D)** Luminol, **(E)** 2-APB, **(F)** AACOF3, **(G)** ETYA, **(H)** Allopurinol, **(I)** GF109203X, **(J)** Safingol, **(K)** Hispidin, and **(L)** U0126 in neutrophils exposed to CpPLC. Negative controls (NC) show NETs and ROS induction in unstimulated neutrophils. Results show means ± SEM. Treatments with statistically significant differences compared to the positive controls (concentration 0 of antioxidants and inhibitors correspond to neutrophils exposed to CpPLC only) are indicated by * (*P* < 0.05) and ** (*P* < 0.01).

Supplementary Table 1 DNAses from *C. perfringens* or *Clostridium* sp. at Uniprot.

Supplementary Table 2 Q8XKM6 peptides detected from bands 1-17 digestion.
