# Supplementary figures and images for: Clostridium perfringens phospholipase C, an archetypal bacterial virulence factor, induces the formation of extracellular traps by human neutrophils
Source: Front Cell Infect Microbiol. 2023 Oct 27;13:1278718. doi: 10.3389/fcimb.2023.1278718 (PMC10641792; doi:10.3389/fcimb.2023.1278718)

## Supplementary Figure 1

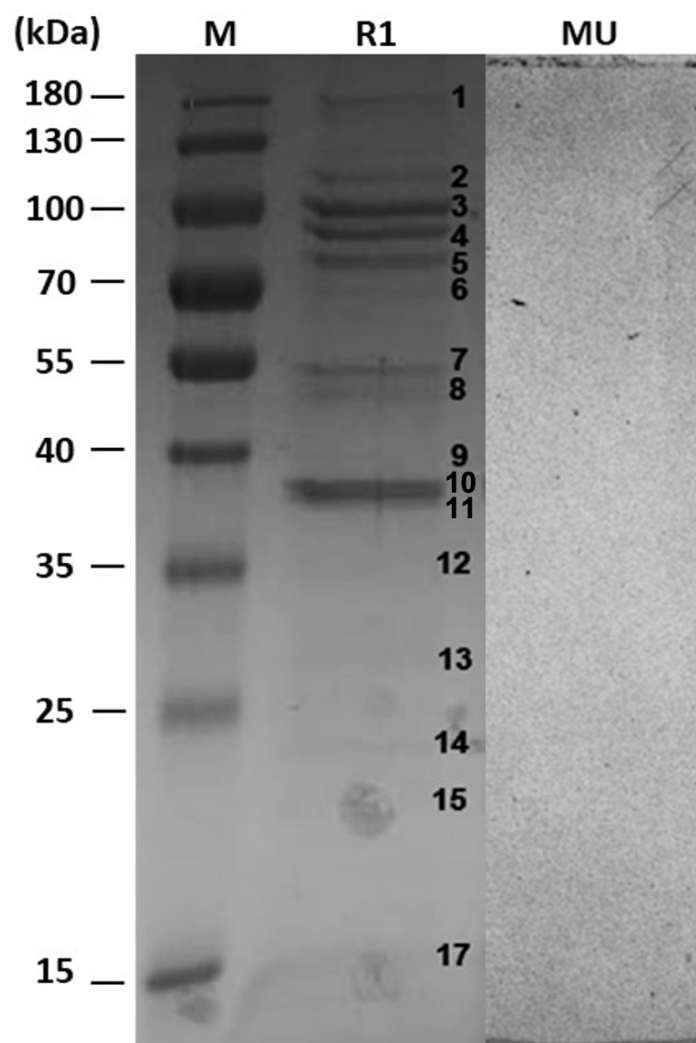

Supplement: Supplementary Figure 1 — SDS-PAGE of C. perfringens secreted proteins at the stationary phase. C. perfringens secreted proteins were separated by SDS-PAGE and stained with Coomassie Brilliant Blue. The bands were cut out for in gel digestion and subsequent peptide analysis by mass spectrometry. [file DataSheet_1.pdf]

### Supplementary Figure 3

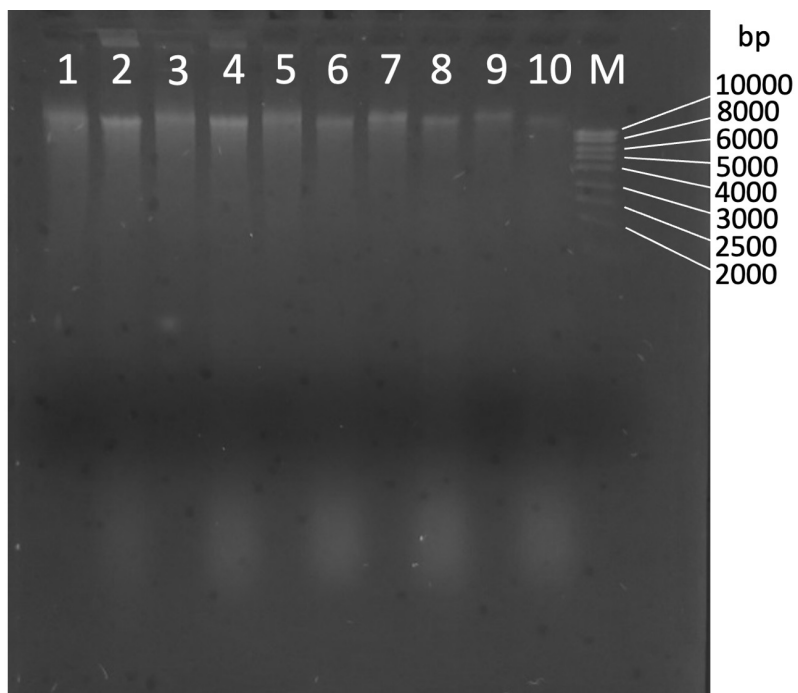

Supplement: Supplementary Figure 3 — C. perfringens secreted proteins degrade DNA. Agarose gel electrophoresis shows the result of calf thymus DNA degradation by C. perfringens secreted proteins. Lanes 1, 3, 5, 7, and 9: calf thymus DNA control 1 µg, 0.5 µg, 0.250 µg, 0.125 µg and 0.0625 µg respectively. Lanes 2, 4, 6, 8, and 10: calf thymus DNA 1 µg, 0.5 µg, 0.250 µg, 0.125 µg, and 0.0625 µg, respectively exposed to the proteins secreted by C. perfringens to the supernatants. M: Thermo Fisher molecular weight marker Mass Ruler High Range DNA Ladder (#SM0393). [file DataSheet_3.pdf]

## Supplementary Figure 4

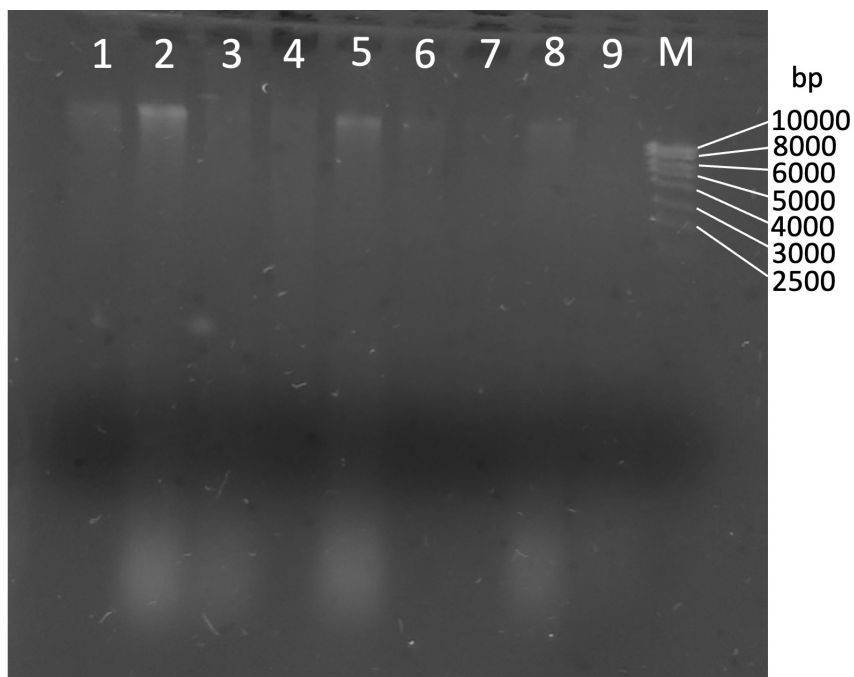

Supplement: Supplementary Figure 4 — CuSO4 inhibits DNA degradation by C. perfringens secreted proteins in a dose-dependent manner. Agarose gel electrophoresis shows the result of calf thymus DNA degradation by C. perfringens secreted proteins. Lanes 1, 4, and 7: calf thymus DNA control 0.250 µg, 0.125 µg, and 0.0625 µg, respectively. Lanes 2, 5, and 8: calf thymus DNA 0.250 µg, 0.125 µg, and 0.0625 µg, respectively, exposed to C. perfringens secreted proteins. Lanes 3, 6, and 9: the same previous set of samples and exposed to CuSO4. M: Thermo Fisher molecular weight marker MassRuler High Range DNA Ladder (#SM0393). [file DataSheet_4.pdf]

# Supplementary Figure 7

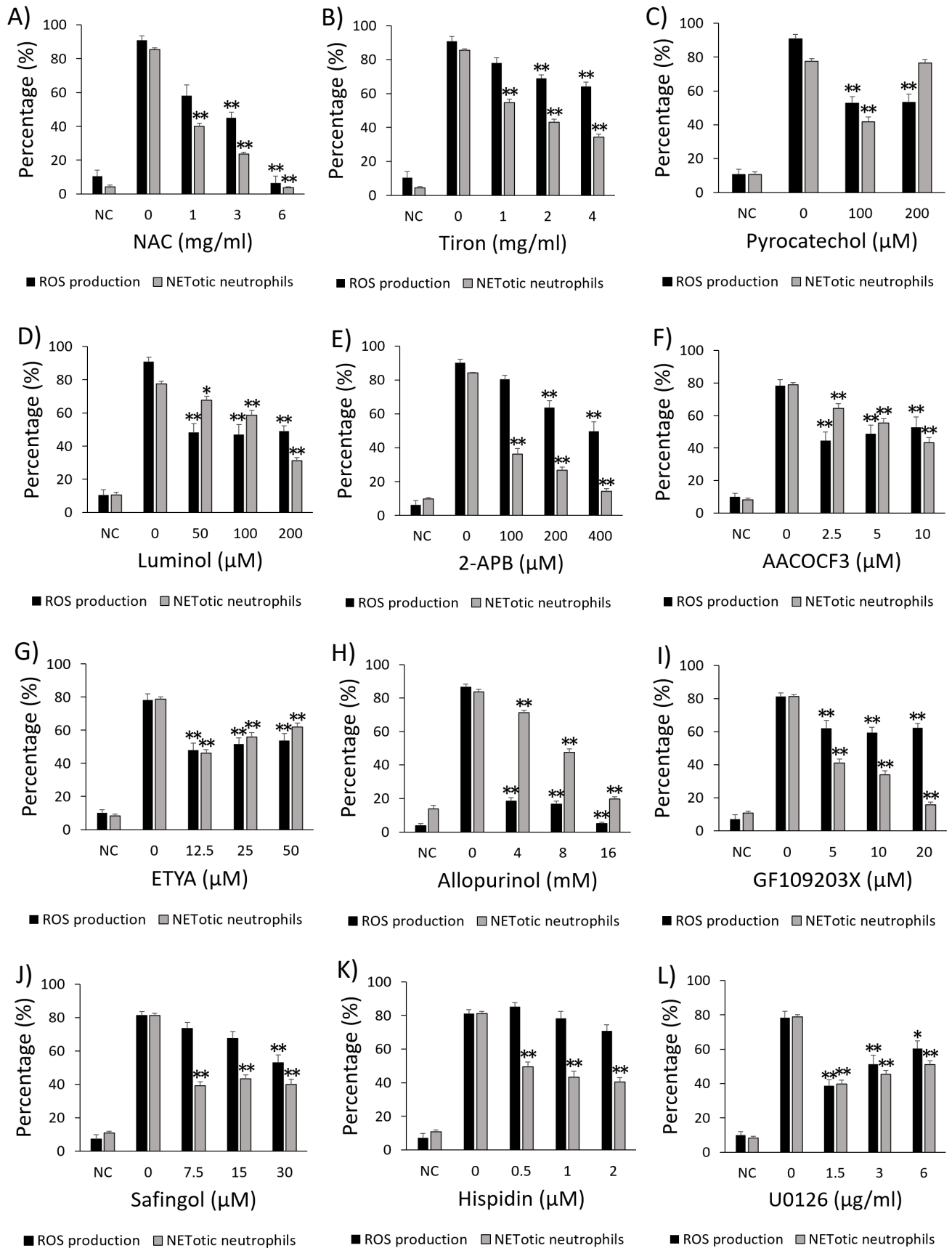

Supplement: Supplementary Figure 7 — Antioxidants and signal transduction pathways inhibitors blocked ROS production and NETs induction by CpPLC. Dose-dependent inhibition of ROS production and NETs formation by (A) NAC, (B) Tiron, (C) Pyrocatechol, (D) Luminol, (E) 2-APB, (F) AACOF3, (G) ETYA, (H) Allopurinol, (I) GF109203X, (J) Safingol, (K) Hispidin, and (L) U0126 in neutrophils exposed to CpPLC. Negative controls (NC) show NETs and ROS induction in unstimulated neutrophils. Results show means ± SEM. Treatments with statistically significant differences compared to the positive controls (concentration 0 of antioxidants and inhibitors correspond to neutrophils exposed to CpPLC only) are indicated by * (P < 0.05) and ** (P < 0.01). [file DataSheet_7.pdf]
