# Supplementary material for: Clostridium perfringens phospholipase C, an archetypal bacterial virulence factor, induces the formation of extracellular traps by human neutrophils
Source: Front Cell Infect Microbiol. 2023 Oct 27;13:1278718. doi: 10.3389/fcimb.2023.1278718 (PMC10641792; doi:10.3389/fcimb.2023.1278718)
Supplement: Supplementary Figure 2 — Sequence alignment of the C. perfringens C8XIF9 bifunctional metallophosphatase/5’-nucleotidase with streptococcal 5´-nucleotidases. Accession codes in the Uniprot database are given on the left. Residues conserved in all sequences are indicated with an asterisk, and positions strictly conserved are indicated with two dots. [file DataSheet_2.pdf]

```
CLUSTAL O(1.2.4) multiple sequence alignment


tr|Q8XIF9|Q8XIF9_CLOPE              MRKLIKPLSV-ATMMFLSLN----------------------------------------
tr|A3CKJ7|A3CKJ7_STRSV              MSKSSLQKTV-ALLSAAALAATVNAVQADENTPAVTANLAPVESSAAEAKPTTAASPTEA
tr|A0A5S4TI37|A0A5S4TI37_STRPY      MKKYFILKSSVLSI-LTSFTLLVTDVQAD-------------------------------
tr|A0A3Q8B6L4|A0A3Q8B6L4_STRSU      MKKKKILLPVMSTLLLAPFV-LAQQVQAAETTTAATTTNQPATTDATAT-----------
                                    * *          :    :

tr|Q8XIF9|Q8XIF9_CLOPE              ------------------------LCFFNGKIVKGE----EISNETKVTILGTSDIHGRF
tr|A3CKJ7|A3CKJ7_STRSV              TATTESTDPSSAISPENAIGDTDALMAMARNVAATEDTKPVEGQTVDVRILATTDLHTNL
tr|A0A5S4TI37|A0A5S4TI37_STRPY      -------------------------------------------QVDVQFLGVNDFHGAL
tr|A0A3Q8B6L4|A0A3Q8B6L4_STRSU      -------------V----------PATSVENVATGETVVPAAAETVEAVIIHTNDVHGRI
                                                                                 ... :: ..*.*  :

tr|Q8XIF9|Q8XIF9_CLOPE              VPWEYSSDTENKS---------GSLSQISTIVKKERNENPNLILVDAGDSIQDNFV----
tr|A3CKJ7|A3CKJ7_STRSV              VNYDYYQD--------KPVETLGLAKTAVLIE-KAKKENPNVLLVDNGDTIQGTPLGTYK
tr|A0A5S4TI37|A0A5S4TI37_STRPY      DNTGTAYTPSGKIPNAGTAAQLGAYMDDAEIDFKQANQDGTSIRVQAGDMVGASPANSAL
tr|A0A3Q8B6L4|A0A3Q8B6L4_STRSU      LEEKNV---------------IGDAKAAAVIEEERA-KVENTIVVDAGDAFQGLPISN-S
                                                          *       *  :   :  . : *: ** .

tr|Q8XIF9|Q8XIF9_CLOPE              ---ETFNKGPHQPMVLGMNKMKYDVWEMGNHEFNFGLDVLKHVTSQFEG-KVLAGN----
tr|A3CKJ7|A3CKJ7_STRSV              AIVDPVEKGEQHPMYAALQALGFEAGTLGNHEFNYGLDYLNRVIETAGM-PLVNAN----
tr|A0A5S4TI37|A0A5S4TI37_STRPY      L--------QDEPTVKVFNKMKFEYGTLGNHEFDEGLDEFNRIMTGQAPDPESTINDITK
tr|A0A3Q8B6L4|A0A3Q8B6L4_STRSU      T--------KGEDRANIMNQVGYDAMAVGNHEFDFGMDQAIKYKETLNF-PLLSAN----
                                               .     :: : ::   :*****: *:*   :             *

tr|Q8XIF9|Q8XIF9_CLOPE              ---------------IY---NDDGTRFMDGYTIIE---------RDGIKIGIIGMDTPMI
tr|A3CKJ7|A3CKJ7_STRSV              ---------------VLDPAT--GKFIYQPYKIIEKTFTDTQGRLTTVKIGVTGIVPPQI
tr|A0A5S4TI37|A0A5S4TI37_STRPY      QYEHEASHQTIVIANVIDKKTNDIPYGWKPYAIKDIAI-----NDKIVKIGFIGVVTTEI
tr|A0A3Q8B6L4|A0A3Q8B6L4_STRSU      TYVNGA--RVFEASTIVDKTP-------------TVV-----GDEFVV---IGVTTPET
                                                   :                               :     *:

tr|Q8XIF9|Q8XIF9_CLOPE              KEFEKPYNIKGIEFRDPVKETKKIIKELDGK-------VDAMIGVMHMGLDNENAISNTG
tr|A3CKJ7|A3CKJ7_STRSV              LNWDKANLEGKVVVRDSVEAIRDIIPEMRKAG------ADITLVLSHSGIGDDKYE---K
tr|A0A5S4TI37|A0A5S4TI37_STRPY      PNLVLKQNYEHYQFLDVAETIAKYAKELQEQ------HVHAIVVLAHVPATSKDGVV--D
tr|A0A3Q8B6L4|A0A3Q8B6L4_STRSU      ATKTHPKNVEGVTFTDPVTEVNKVIDEVEARALADNRVVYKNYIILAHLGVDSTTPVEWRG
                                                 . * .    .   *:           .  : : *    .

tr|Q8XIF9|Q8XIF9_CLOPE              VT--------DIANQCPELTAIVGGHMHKLVKNEVV-----------------NGVIITE
tr|A3CKJ7|A3CKJ7_STRSV              GE----ENEGYQIASLPGVDAVVTGHSHAEFPSGNGTGFYEKYPGVDGINGKINGTPVTM
tr|A0A5S4TI37|A0A5S4TI37_STRPY      HEMATVMEKVNQIYPEHSIDIIFAGHNHQYTNGTIGKTR--------------IVQALSQ
tr|A0A3Q8B6L4|A0A3Q8B6L4_STRSU      STLAEALSKNSKLA--GKRVIVIDGHSHTVEATTYGENV--------------T---YNQ
                                                         :. ** *                              .

tr|Q8XIF9|Q8XIF9_CLOPE              PGKYGQAVSKIDLTFKKENGKNVLKNKNADTI--SVANVESDKEIEDLLKPFHEELRKDA
tr|A3CKJ7|A3CKJ7_STRSV              AGKYGDHLGVIDLKLNYTDGKWKVTDSKGSIRKVDTKSNVADQRVIDIAKE------SHQ
tr|A0A5S4TI37|A0A5S4TI37_STRPY      GKAYADVRGTLDTDTNDFM-------------------TTPSANVVAVAPGIKTENSDIK
tr|A0A3Q8B6L4|A0A3Q8B6L4_STRSU      TGSYLNNIGKVTLKSDKLLGEASLISA------ADTKNVTPNAKIAALVDEIKAKYEAEN
                                       * :  . :    .                         . .:  :

tr|Q8XIF9|Q8XIF9_CLOPE              ------NS----VIGRLEGVNMVDEDYIKGIPTIHIEDTPLIDFFHEVGKYYSKADVIA-
tr|A3CKJ7|A3CKJ7_STRSV              GTINY--------VRQQVGITT--A--PITSYFSLVKDDPSVQIVNNAQLWYAKQELAGT
tr|A0A5S4TI37|A0A5S4TI37_STRPY      AIINHANDIVKTVTERKIGTATNSS--TISKTENIDKESPVGNLVTTAQLTIAKKTFP-T
tr|A0A3Q8B6L4|A0A3Q8B6L4_STRSU      AQVVIENNP--------VELNG-------DRSNVRVRETNLGNAVTDAIYAYGQTGFSNK
                                                                    .:    : .  .    .:  .

tr|Q8XIF9|Q8XIF9_CLOPE              ----LSI-----------DND---KAKLNVGDIKKDIAYNYRYTGGEISVYEVTGKDLK
tr|A3CKJ7|A3CKJ7_STRSV              PEANLPILSAAAPFKAGTRGDATAYTDIPAGPIAIKNVADLYL-YDNVTAILKVNGAQLK
tr|A0A5S4TI37|A0A5S4TI37_STRPY      VDF-------AMTNNGGIRSDLVVKND---RTITWGAAQAVQP-FGNILQVIQMTGQHIY
tr|A0A3Q8B6L4|A0A3Q8B6L4_STRSU      TSL-------AVTNGGGLRATI--AKD---QPVTKGDIIAVLP-FGNIVSQITVTGQQIY
                                                              .     :            ..      :.* .:

tr|Q8XIF9|Q8XIF9_CLOPE              KYMEWAAGYFNTLNPGDITPSFNPKRRASKYST--NDMFGGITYKIDLREKEGNRI----
tr|A3CKJ7|A3CKJ7_STRSV              EWLEMSAGQFNTIDPNNNQPQ---NLVNTDYRTYNFDVIDGVTYEFDITQPNKYDREGKL
tr|A0A5S4TI37|A0A5S4TI37_STRPY      DVLNQQYDENQ---------------------TYFLQMSGLTYTYTDNDPKNSDT----
tr|A0A3Q8B6L4|A0A3Q8B6L4_STRSU      DMFTKSLSSTLQVNPETGEMLLDENGMPLFEASGGFLHISGANVFYDPTLPVEERVL---
                                    . :    .                              :.* .

tr|Q8XIF9|Q8XIF9_CLOPE              -------KDVKYKDGRELKDTDVLKLGMNSYRLGQLQGKGGIFEGKEFKKLWDSKTAYGE
tr|A3CKJ7|A3CKJ7_STRSV              ANPNASRVRNLKYQGKEIDPNQEFIVVTNNYRSNGN--FPGVREA-SLNRLLNLE-----
tr|A0A5S4TI37|A0A5S4TI37_STRPY      ---PFKIVKVYKDNGEEINLTTTYTVVVNDFLYGGGDGFSAFKKA---------------
tr|A0A3Q8B6L4|A0A3Q8B6L4_STRSU      ---LIGILNPETGEYDALDLEKTYYLATNDFLAAGGDGYTMLGGA---------------
                                                 :   :.      :  *.:          .  .

tr|Q8XIF9|Q8XIF9_CLOPE              EEGTIRNLAIDYIKNVKNGLINTKKQNNWCLLGIDPN--------SENYKKVRDLVNSGE
tr|A3CKJ7|A3CKJ7_STRSV              ----NRQAIINYILAVK--NINPSADQNWHFADTIKGLDLRFLTAD----KAKNLIGTDG
tr|A0A5S4TI37|A0A5S4TI37_STRPY      ----------------K--LIGAINTDTEAFITYITNLEASGKTVNATIKGVKNYVTSNL
tr|A0A3Q8B6L4|A0A3Q8B6L4_STRSU      ----------------R--EEGPSMD--SVFAEYLKTADLSAYEVVNPYSR---II--PV
                                                    :    .        :                        :
```

```
tr|Q8XIF9|Q8XIF9_CLOPE              ---LKIPTSEDGKYTNIASINEKDLPSDNNTSKENEENINLDSINNKNNNKDQEVNEESK
tr|A3CKJ7|A3CKJ7_STRSV              DIVYLAASAQEGF-GEYKFVY----------VAPKTEPVPIEQ-----------------
tr|A0A5S4TI37|A0A5S4TI37_STRPY      --ESSTKVNSAGK-HSIISKV---FRNRDGNIVSS-EII---------------------
tr|A0A3Q8B6L4|A0A3Q8B6L4_STRSU      --NSSIDTDEDGY-PDFIEIL---LDTDPENPASNPETVPAEN-----------------
                                             . *   .                  . * :

tr|Q8XIF9|Q8XIF9_CLOPE              KDVPKVEENIEKQKNNKENNSNGDNTLVKEDSKS-KEVNEKNNEQNNIEEVSKKENKLPN
tr|A3CKJ7|A3CKJ7_STRSV              PSSPT---------IAVEAANLQH-S--KVDFPVLTAVDPSTNKQA--SHRQAGAESLPV
tr|A0A5S4TI37|A0A5S4TI37_STRPY      ----S---------DLLTSTENT--------NNSLGKKETT----T--NKNTISSSTLLI
tr|A0A3Q8B6L4|A0A3Q8B6L4_STRSU      TDSPS---------NQVQNTSATD-KKVPVDSPKVGDKKPD--VAS--PVKTTKAGVLPN
                                        .               .                 .                  *

tr|Q8XIF9|Q8XIF9_CLOPE              TGSPIGAEAMSQIGMLLLGAGVILKKKNKK--
tr|A3CKJ7|A3CKJ7_STRSV              TGEKTSSLGLLG-LVMTGLAGIFTFKKRERQ-
tr|A0A5S4TI37|A0A5S4TI37_STRPY      TGDNYKMSPIMTILALISLGGLNAFIKERKS-
tr|A0A3Q8B6L4|A0A3Q8B6L4_STRSU      TGDQMNLTL--SL-FGLGLAGLAMAVGRRKEN
                                    **.                .*:     ..:
```
