# Supplementary material for: Clostridium perfringens phospholipase C, an archetypal bacterial virulence factor, induces the formation of extracellular traps by human neutrophils
Source: Front Cell Infect Microbiol. 2023 Oct 27;13:1278718. doi: 10.3389/fcimb.2023.1278718 (PMC10641792; doi:10.3389/fcimb.2023.1278718)
Supplement: Supplementary Figure 5 — Amino acid sequence of the DNAse present in the C. perfringens secretome. The amino acid sequence of the cell-wall anchored DNAse (Uniprot Accession number Q8XKM6) is shown. The shadowed segments correspond to the peptides identified by tandem mass spectrometry after in gel digestion of C. perfringens secreted proteins. [file DataSheet_5.pdf]

Supplementary Figure 5

1 MKKDYKILIT KALSLSMVGS LFTSYPTFAK EIKTQEGDLI ISEYLHGPSN SK**AIEIFNGT GEG**INLSNYD LAVYSNGK**YE**

81 **GSPVSQSLED K**IIESGETYV IYNNQGKDEK **FTEVINDLEN KLGVGSQ**TVG FNGDDVVLLR KNTGSGYEII DSFGAKTEKD

161 KKFYDSKFIS ARRKSEIKDG ENNIDKTPFD V**SVQWDVDTE NLYDDLGK**HD ISWSEGENPS GKVLKVKEAR NK**NLGEEVTT**

241 **R**GVVTFNDRN KTLHIQDETG AIAISNFK**SG VDFGAITKGN K**IEISGTLDN FNGLLQVQAT DIKVLGDLGM PDPKLVTIKE

321 LK**ESNFDSHY IELKNTVVDL EAKTLTQG**ED VLDIYFIPSG LEVKTGDLVD VKGVIGRFND KVQLYGSSAE FTKIVEDNES

401 PVITHKKIEK ANINEDLNIE AKVSDNNKLE EVSISFKGKE DTEFKKVVLK EEDGIFKALI PKEDLKA**SGM EYYIEASDGK**

481 NISRVPESGV YAFQVVDEDL SGPEVK**NVLP KENSSVGENR RPVISGEFID NSGVNVESVK** IK**LDNEDITK RA**KITEAGFS

561 YEIEKDLEDG EHRVEVSVSD SLGNNRVKEW KFRVGKINHY YGQLHSHTN**I SDGTGSLEDA YKWARDEGNA DYFAVTDHSN**

641 **WFDNDTEAN**I NDGSMSK**AWT NAQNISDKYN D**DGNFVAMYG YEMTWSGSTG GWGHINTFNT PGFETR**KNSD MNLK**NYYNTI

721 SQLPESVSQL NHPGKTFGDF ADFGFYSEGA DK**VVNLIEVG NGEG**PVRGSG YFPSYEYYTR ALDKGWHVAP TNN**QDNHKGK**

801 WLTANDARTI ILSEENSRDA LYKAMNK**KQV YSSEDK**NMTI DYTVNNQIMG SNLGEVEDLD FNIEINDEDK EDTIKKVSII

881 **ANGGVEVISK** EFNSNKVSWN FKLKPEYSYY YVKVVQGDQD IAVTAPVWIG EN**VNVGLNEL KTDKDMLLVG DEAKLSIEVY**

961 **NNSSER**LNNI KVEFFNGEIS DEKKIGEEII ESLEGNSLK**E SSITWQPER**A GEFTIYAK**AT ISINGTDKTF TKSSK**IEVVN

1041 EGDVYK**VMID GAHANQYVTG N**YAGKIDAFE KLLTENDCIP IINKEEITEK SLENVDLLVI TDPQGID**EPK YEVYK**SNFTD

1121 SEIDAIGKYM DKGGNIIITS RADYKDGVGE YSNGAQLNPI LEKINSELRV NDDQVAD**YEV NEGQQFRLML NK**YSSPNFNL

1201 VEGLGEEDKF SFYSGSSVVL KDGAKGEKVD FLVSGHESTG TDDSDNQGDN VPLEKGQVNV LAVEELSNGG KVAVAGSTFF

1281 SNFEIDG**TNA ESKSNSKVTK** NIINWMLPEK ELEKLTIKEF REDKNNDGEP DRLGEEYVLE GIVT**AQSEAV EPK**NAFFEVI

1361 YIQDETGGIN VFGVSNTPVK VGQKVR**VKGR VEAYQGE**FEI QISDESADLE IIDENINEVS PKEMSTGDSM LHENEGWLTK

1441 VTGKVVNMDD SNLYLDDGSG VSRIYVEGYI WDGINENMKG KWDPRIKVGD TVSAIGLSSE DPEGNRLRVR **NTGEIVLQ**EE

1521 ENLGVINTEI TSDKNEIK**EN EKISLLAK**VE NRTKEVLENV TLK**IFANNGE NEVLLKEEKI DSLGANESK**E LTFEHAFELE

1601 GRYSIGIKLF DSEGNEIKSK NKEFSLVVLK EINGGDSDNN GDANNGGDSN NGSGNDSGEE NKPGTDKPNT EKPEELPNTG

1681 NRMNANMLMG FGALYLALGF YMVSKRKKVR
