# Supplementary material for: Clostridium perfringens phospholipase C, an archetypal bacterial virulence factor, induces the formation of extracellular traps by human neutrophils
Source: Front Cell Infect Microbiol. 2023 Oct 27;13:1278718. doi: 10.3389/fcimb.2023.1278718 (PMC10641792; doi:10.3389/fcimb.2023.1278718)
Supplement: Supplementary Figure 6 — Sequence alignment of the C. perfringens Q8XKM6 DNAse with streptococcal nucleases. Accession codes in the Uniprot database are given on the left. Residues conserved in all sequences are indicated with an asterisk, and positions strictly conserved are indicated with two dots. [file DataSheet_6.pdf]

CLUSTAL O(1.2.4) multiple sequence alignment

```
tr|Q8XKM6|Q8XKM6_CLOPE              MKKDYKILI--------TKALSLSMVG----------SLFTSYPTFAKEIKTQEGDLIIS
tr|A3CPM7|A3CPM7_STRSV              ------------------------------------------------------------
tr|A0A455ZLU4|A0A455ZLU4_STRP1      ---------------------------------------------------------MIN
tr|Q6X5T7|Q6X5T7_STRSU              MKIRNRSLFYTVGSVAVTAGLLLSLATSPIPSVHATEVAIENYPSLA----ITKSEVTIQ


tr|Q8XKM6|Q8XKM6_CLOPE              EYLHGPSNSKAIEIFNGTGEGINLSNYDLAVYSNGKYEGSPVSQSLEDKIIESGETYVIY
tr|A3CPM7|A3CPM7_STRSV              ------------------------------------------------------------
tr|A0A455ZLU4|A0A455ZLU4_STRP1      KKCIIPVSLLTLA-----------------------------------I-----------
tr|Q6X5T7|Q6X5T7_STRSU              GYLIAPLNSSGTAFDATNKT-----NLAL---------GASMDTAADTI-----------


tr|Q8XKM6|Q8XKM6_CLOPE              NNQGKDEKFTEVINDLENKLGVGSQTVGFNGDDVVLLRKNTGSGYEIIDSFGAKTEKDKK
tr|A3CPM7|A3CPM7_STRSV              ------------------------------------------------------------
tr|A0A455ZLU4|A0A455ZLU4_STRP1      ---------------------------TLTSVEEVTSRQNLTYANEIVTQRP--------
tr|Q6X5T7|Q6X5T7_STRSU              ---------------------------PIQLKEPLRSQFNLVNHPELVGKLVR-------


tr|Q8XKM6|Q8XKM6_CLOPE              FYDSKFISARRKSEIKDGENNIDKTPFDVSVQWDVDTENLYDDLGKHDISWSEGENPSGK
tr|A3CPM7|A3CPM7_STRSV              ------------------------------------------------------------
tr|A0A455ZLU4|A0A455ZLU4_STRP1      ---------KRESVISDKSNFPVISPYL--ASV---------DFGERKTPLPTPDK---G
tr|Q6X5T7|Q6X5T7_STRSU              ------ITGTSDTYMKR----AGIKPATAIEIV---------DSSSTNVQPPTSENTATK


tr|Q8XKM6|Q8XKM6_CLOPE              V--L---KVKEARNKNLGEEVTTRGVVT--FND-RNKTLHIQDETGAIAISNFKSGVDFG
tr|A3CPM7|A3CPM7_STRSV              ------------------------------------------------------------
tr|A0A455ZLU4|A0A455ZLU4_STRP1      VKVTTEQSIAQVRKGPEERPYTVTGKITSVINGWGGYGFYIQDSEGIGL-YV-YPQKDL-
tr|Q6X5T7|Q6X5T7_STRSU              PSDLVSTPIATVRSGAQGTEYTVSGKIISLVNGWGGNGFYLQGSDGAGI-YI-YPGAAL-


tr|Q8XKM6|Q8XKM6_CLOPE              AITKGNKIEISGTLDNFNGLLQVQATDIKVLGDLGMPDPKLVTIKELKESNFDSHYIELK
tr|A3CPM7|A3CPM7_STRSV              -------------------------MKKQVSYKQLFPTVVLLAAFLS-------LLFSVQ
tr|A0A455ZLU4|A0A455ZLU4_STRP1      GYSKGDIVQLTGTLTRFKGDLQLQQVTAHKKLELSFPTSVKEAVISELETTTPSTLVKLS
tr|Q6X5T7|Q6X5T7_STRSU              GYQLGDTVQLTGTLGEYKGELQLTTVSNHKAISENFNTPITETNIAQLATQAQATLVSLK
                                                                :   .  :      :             ..:.

tr|Q8XKM6|Q8XKM6_CLOPE              NTVVDLEAKTLTQGEDVLDIYFIP-----------------SG---------LEVKTGDL
tr|A3CPM7|A3CPM7_STRSV              ---------PVGAEEAVSSSTSDA-------------AALTENPAVSDASVASQS-----
tr|A0A455ZLU4|A0A455ZLU4_STRP1      ---------HVTVGELSTDQYNNTSFLVRDDSGKSIVVHIDHRTGVKGADVVTKISQGDL
tr|Q6X5T7|Q6X5T7_STRSU              ---------NLTVGDIQSDSYQNSTFTVTDSEGQTVDVRLDSRTGIKTADLLNRINKGDK
                                              :   :   .                                  .

tr|Q8XKM6|Q8XKM6_CLOPE              VDVKGVIGRFNDKVQLYGSSAEFTKIVEDNESPVITHKKIEKANINEDLNIEAKVSDNNK
tr|A3CPM7|A3CPM7_STRSV              ----ESSSAESGESR--ATSEEA-------------------------------------
tr|A0A455ZLU4|A0A455ZLU4_STRP1      INLTAILSIVDGQLQLRPFSLEQ-------------------------------------
tr|Q6X5T7|Q6X5T7_STRSU              INLTAILSTYNGKIQLKPFDLSH-------------------------------------
                                           .  ..: :    . .

tr|Q8XKM6|Q8XKM6_CLOPE              LEEVSISFKGKEDTEFKKVVLKEEDGIFKALIPKEDLKASGMEYYIEASDGKNISRVPES
tr|A3CPM7|A3CPM7_STRSV              ------------------------------------------------------------
tr|A0A455ZLU4|A0A455ZLU4_STRP1      ------------------------------------------------------------
tr|Q6X5T7|Q6X5T7_STRSU              ------------------------------------------------------------


tr|Q8XKM6|Q8XKM6_CLOPE              GVYAFQVVDEDLSGPEVKNVLPKENSSVGENRRPVISGEFIDNSGVNVESVKIKLDNEDI
tr|A3CPM7|A3CPM7_STRSV              ----VGKADQAPAAAQAATSGPEVVPNVGTIQGESQASPYGD-KEVQVSNVVVTK-----
tr|A0A455ZLU4|A0A455ZLU4_STRP1      ----LEVV--KKVTSSNSDASSRNIVKIGEIQGASHTSPLLK-KAVTVEQVVVTY-----
tr|Q6X5T7|Q6X5T7_STRSU              ----FEVI--EKATTEA-GLGKTEAVTVGRIQGASHQSPLVN-QSVMLKNVVVTY-----
                                        .          .          .:*  :     .   . . * :..* :.

tr|Q8XKM6|Q8XKM6_CLOPE              TKRAKITEAGFSYEIEKDLEDGEHRV-----------EVSVSDSLG------NNRVKEWK
tr|A3CPM7|A3CPM7_STRSV              ------T-DRYGFYVQDVTPDGNSRTSDALYVVSK-EKVDVGDKLSLEGRVKEGYMEELS
tr|A0A455ZLU4|A0A455ZLU4_STRP1      -----LD-DSTHFYVQDLNGDGDLATSDGIRVFAKNAKVQVGDVLTIS-----GEV-EEF
tr|Q6X5T7|Q6X5T7_STRSU              -----VT-SANNFYVQDVTPDGDTKTSDGINIFTDKLKTNVKGRRSCHYRR--DEVERIP
                                                : ::.   **:  .           :..* .          . : .

tr|Q8XKM6|Q8XKM6_CLOPE              FRVGK------INHYYGQLHSHTNISDGTGSLEDAYKWARDEGNADYFAVTDHSNWFDND
tr|A3CPM7|A3CPM7_STRSV              VRQGQTFNKPSDSLTVTMLVASKVIKEGKADLPAPVDIV--ANM--------PKDTVDN-
tr|A0A455ZLU4|A0A455ZLU4_STRP1      FGRGY-EERKQTDLTITQIVAKAVTKTGTAQVPSPLVLGKDRIA--------PANIIDND
tr|Q6X5T7|Q6X5T7_STRSU              RARGY-AERDKTDLTITQIRATEVTVDGTAPVPSPIVLGLDRTI--------PADIIDND
                                       *        .     : :      *.. :                      : .**

tr|Q8XKM6|Q8XKM6_CLOPE              TEANIND-GSMSKAWTNAQNISDKYNDDGNFVAMYGYEMTWSGSTGGWGHINTFNTPGFE
tr|A3CPM7|A3CPM7_STRSV              DINNYQPQSEALDYWESLEGMLTTVKRPRVLGPQY-------------------------
tr|A0A455ZLU4|A0A455ZLU4_STRP1      GLRVFDPEEDAIDYWESMEGMLVAVDDAKILGPMK-------------------------
tr|Q6X5T7|Q6X5T7_STRSU              GLAQFDPEQDALDFWESVEGMVVAVDDAKILGPLK-------------------------
                                         :   .  . * . :.:    .    :
```

```
tr|Q8XKM6|Q8XKM6_CLOPE              TRKNSDMNLKNYYNTISQLPESVSQLNHPGKTFGDFADFGFYSEGADKVVNLIEVGNGEG
tr|A3CPM7|A3CPM7_STRSV              ------------RGDIYVLPEGYQALPLN-----NIGGLNLRPNAQNTATIPVYVG-NKF
tr|A0A455ZLU4|A0A455ZLU4_STRP1      ------------NKEIYVLPGS-STRPLN-----NSGGVLLPANSYNTDVIPVLFKKGKQ
tr|Q6X5T7|Q6X5T7_STRSU              ------------NKEIYVTPAT-SQLPLN-----NVGGVNLRPEGNNTNIIPLLLKNGKQ
                                               *   *   .          : ... :  :. :.    : .  .:

tr|Q8XKM6|Q8XKM6_CLOPE              PVRGSGYFPSY----EYYTRALDKGWHVAPTNNQDNHKGKWLTANDARTIILSEENSRDA
tr|A3CPM7|A3CPM7_STRSV              IAKAKDYFNGDVVGVVTYRGKVYK---LEPTQLPDLVDGGLQR-----------------
tr|A0A455ZLU4|A0A455ZLU4_STRP1      IIKAGDSYKGRLAGPVSYSYGNYKVFVDDSKNMPSLMDGHLKP-----------------
tr|Q6X5T7|Q6X5T7_STRSU              IVKSGDYFIGRIAGPVTYSYTNYKVYVDDS-TLPTLHEGATKP-----------------
                                      :. . : .       *     *             .*

tr|Q8XKM6|Q8XKM6_CLOPE              LYKAMNKKQVYSSEDKNMTIDYTVNNQIMGSNLGEVEDLDF----NIEINDEDKEDTIKK
tr|A3CPM7|A3CPM7_STRSV              -----QVSPIYPSEDKLTVASYNIENFSANAKKGETPEEKVTRIANSFINEIHSPDIITL
tr|A0A455ZLU4|A0A455ZLU4_STRP1      -----EKTNLQKDLSKLSIASYNIENFSANPSS--TKDEKVKRIAESFIHDLNAPDIIGL
tr|Q6X5T7|Q6X5T7_STRSU              -----ETTTIIPNDDKLTIASYNIENFSANSKS--TSDAKVQRIAKSFVSDLHSPDVIGL
                                         : . :  . .*    .*.::*   . .   . : ..    :  : : .  * *

tr|Q8XKM6|Q8XKM6_CLOPE              VSIIANGGVEVI---SKEFNSNKVSWNFKL--KPEYSYY---------------------
tr|A3CPM7|A3CPM7_STRSV              IEVQDENGSVNDGTTSGVKSGEKLAARIKELGGKTYKYTEVAPLDGQDGGKPGSNIRVAF
tr|A0A455ZLU4|A0A455ZLU4_STRP1      IEVQDNNGPTDDGTTDATQSAQRLIDAIKKLGGPTYRYVDIAPENNVDGGQPGGNIRTGF
tr|Q6X5T7|Q6X5T7_STRSU              IEVQDNNGATNDGTTDASKSAERLIAAIQAAGGPTYTYVDIAPENNKDGGQEGGNIRVGF
                                    :.:  :.*       .   ..:::   ::      * *

tr|Q8XKM6|Q8XKM6_CLOPE              -----YVKVVQGDQDIAVTAPVWIGENVNVGLNELKTDKDMLLVGDEAKLSIEVYNNSSE
tr|A3CPM7|A3CPM7_STRSV              LYNPNRVKLVEKDAGNSDEAASFSGGHLVKNPARIEPT----------------------
tr|A0A455ZLU4|A0A455ZLU4_STRP1      LYQPERVSLSDKPKGGARDALTWVNGELNLSVGRIDPT----------------------
tr|Q6X5T7|Q6X5T7_STRSU              LYNSKRVSLSDKPIGTATQAVAWENGELNLSLGRIDPT----------------------
                                          *.: :   . :  *  : . .:  .  .:.

tr|Q8XKM6|Q8XKM6_CLOPE              RLNNIKVEFFNGEISDEKKIGEEIIESLEGNSLKESSITWQPERAGEFTIYAKATISING
tr|A3CPM7|A3CPM7_STRSV              ------------------------------------NPAFTKVRK---------------
tr|A0A455ZLU4|A0A455ZLU4_STRP1      ------------------------------------NAAWKDVRK---------------
tr|Q6X5T7|Q6X5T7_STRSU              ------------------------------------NPAWAAVRK---------------
                                                                        . ::   *

tr|Q8XKM6|Q8XKM6_CLOPE              TDKTFTKSSKIEVVNEGDVYKVMIDGAHANQYV--TGNYAGKIDAFEKLLTENDCIPIIN
tr|A3CPM7|A3CPM7_STRSV              -------SLAAEFEFKGQHIVVIA--NHLKSKIGDDAVY-GSA------------QPAVQ
tr|A0A455ZLU4|A0A455ZLU4_STRP1      -------SLAAEFIFQGRKVVVVA--NHLNSKRGDNALY-GCV------------QPVTF
tr|Q6X5T7|Q6X5T7_STRSU              -------TLAAEFVFKGEKVVVLA--NHLNSKRGDNGLY-GKI------------QPVSF
                                           :   *.  :*    *:    * :.     . * *               *

tr|Q8XKM6|Q8XKM6_CLOPE              KEEITEKSLENVDLLVITDPQGID--EPKYEVYKSNFTDSEIDAIGKYMDKGGNIIITSR
tr|A3CPM7|A3CPM7_STRSV              HTQAARIEQA--KILNSFVQEGLRQNPNLKFVLTGDFNDFEFSETAKALAGNELINLMQE
tr|A0A455ZLU4|A0A455ZLU4_STRP1      KSEQRRHVLA--NMLAQFAKEGAK--HQANIVMLGDFNDFEFTKTIQLIEEGDMVNLVSR
tr|Q6X5T7|Q6X5T7_STRSU              KSEEKRHILA--QTIADFTKAGLAQNPNANIVMLGDFNDYEFTKTIEILEAGGMANLVSR
                                    : :  .      . :      *         *  .:*.* *:    : :  .    : ..

tr|Q8XKM6|Q8XKM6_CLOPE              ADYKDGVGEYSNGAQL--NPILEKINSELRVNDDQVADYEVNEGQQFRLMLNKYSSPNFN
tr|A3CPM7|A3CPM7_STRSV              HDAADRYSYFYRGSNQSLDNIFISKN-----------------------LAGKAVFAPVH
tr|A0A455ZLU4|A0A455ZLU4_STRP1      HDISDRYSYFHQGNNQTLDNILVSRH-----------------------LLDHYEFDMVH
tr|Q6X5T7|Q6X5T7_STRSU              HDASDRFSYFYNGNNQSLDNMLVSTN-----------------------LLERYAFDMVH
                                     *  *  . : .* :   : :: . :                      :  :     .:

tr|Q8XKM6|Q8XKM6_CLOPE              LVEGLGEEDKFSFYSGSSVVLKDGAKGEKVDFLVSGHESTGTDDSDNQGDNVPLEKGQVN
tr|A3CPM7|A3CPM7_STRSV              INASFMEEHGRASDHDPVVVQ--------LDFSKDV-AASTSDSSQPS-----QSTGQSS
tr|A0A455ZLU4|A0A455ZLU4_STRP1      VNSPFMEAHGRASDHDPLLLQ--------LSFSKEN-DKAESSKQSV-------------
tr|Q6X5T7|Q6X5T7_STRSU              VNSAFMEEHGRAFDHDPLLVQ--------LDVTKAQ-EPTQPEPSDKQ----TDDSGTVN
                                    :   : * .  :   .  ::         :..       :  . ..

tr|Q8XKM6|Q8XKM6_CLOPE              VLAVEELSNGGKVAVAGSTFFSNFEIDGTNAESKSNSKVTKNIINWMLPEKELEKLTIKE
tr|A3CPM7|A3CPM7_STRSV              VAAEQGAPSQ-----TNPPSSN--------------------------------------
tr|A0A455ZLU4|A0A455ZLU4_STRP1      ------------------------------------------------------------
tr|Q6X5T7|Q6X5T7_STRSU              NSDDNGTTNN-----NKPTNLSTFNQTAVNADDRS-------------------------

tr|Q8XKM6|Q8XKM6_CLOPE              FREDKNNDGEPDRLGEEYVLEGIVTAQSEAVEPKNAFFEVIYIQDETGGINVFGVSNTPV
tr|A3CPM7|A3CPM7_STRSV              ------------------------------------------------------------
tr|A0A455ZLU4|A0A455ZLU4_STRP1      ------------------------------------------------------------
tr|Q6X5T7|Q6X5T7_STRSU              ------------------------------------------------------------

tr|Q8XKM6|Q8XKM6_CLOPE              KVGQKVRVKGRVEAYQGEFEIQISDESADLEIIDENINEVSPKEMSTGDSMLHENEGWLT
tr|A3CPM7|A3CPM7_STRSV              ------------------------------------------------------------
tr|A0A455ZLU4|A0A455ZLU4_STRP1      ------------------------------------------------------------
tr|Q6X5T7|Q6X5T7_STRSU              ------------------------------------------------------------

tr|Q8XKM6|Q8XKM6_CLOPE              KVTGKVVNMDDSNLYLDDGSGVSRIYVEGYIWDGINENMKGKWDPRIKVGDTVSAIGLSS
tr|A3CPM7|A3CPM7_STRSV              ------------------------------------------------------------
```

```
tr|A0A455ZLU4|A0A455ZLU4_STRP1      ------------------------------------------------------------
tr|Q6X5T7|Q6X5T7_STRSU              ------------------------------------------------------------

tr|Q8XKM6|Q8XKM6_CLOPE              EDPEGNRLRVRNTGEIVLQEEENLGVINTEITSDKNEIKENEKISLLAKVENRTKEVLEN
tr|A3CPM7|A3CPM7_STRSV              ------------------------------------------------------------
tr|A0A455ZLU4|A0A455ZLU4_STRP1      ------------------------------------------------------------
tr|Q6X5T7|Q6X5T7_STRSU              ------------------------------------------------------------

tr|Q8XKM6|Q8XKM6_CLOPE              VTLKIFANNGENEVLLKEEKIDSLGANESKELTFEHAFELEGRYSIGIKLFDSEGNEIKS
tr|A3CPM7|A3CPM7_STRSV              ------------------------------------------------------------
tr|A0A455ZLU4|A0A455ZLU4_STRP1      ------------------------------------------------------------
tr|Q6X5T7|Q6X5T7_STRSU              ------------------------------------------------------------

tr|Q8XKM6|Q8XKM6_CLOPE              KNKEFSLVVLKEINGGDSDNNGDANNGGDSNNGSGNDSGEENKPGTDKPNTEKPEELPNT
tr|A3CPM7|A3CPM7_STRSV              ------------------------------------------QTGQGQTASSKKKGLPKT
tr|A0A455ZLU4|A0A455ZLU4_STRP1      ----------------------------------------------KAKKTSKGKLLPKT
tr|Q6X5T7|Q6X5T7_STRSU              -----------------------------------GATDKRQTTVTPTANNSQKKILPKT
                                                                                  ..: : **:*

tr|Q8XKM6|Q8XKM6_CLOPE              GNRMNANMLMGFGALYLALGFYMVSKRKKVR
tr|A3CPM7|A3CPM7_STRSV              GQNTSGW--AVLGLTLLMFAFTLK-KRSRN-
tr|A0A455ZLU4|A0A455ZLU4_STRP1      GDSLVYV-ITLLGTASLLVPILLLTKGKKES
tr|Q6X5T7|Q6X5T7_STRSU              GGETSFV-LITIGLVFLSAC--L-VKKQKES
                                    *          :*   *     :  * .:
```
