# Supplementary material for: Clostridium perfringens phospholipase C, an archetypal bacterial virulence factor, induces the formation of extracellular traps by human neutrophils
Source: Front Cell Infect Microbiol. 2023 Oct 27;13:1278718. doi: 10.3389/fcimb.2023.1278718 (PMC10641792; doi:10.3389/fcimb.2023.1278718)
Supplement: Supplementary Table 1 — DNAses from C. perfringens or Clostridium sp. at Uniprot. [file Table_1.pdf]

Supplementary Table 1. DNAses from *Clostridium perfringens* or *Clostridium sp.* at the Uniprot database.

Accession (Uniprot)	Description	Avg. Mass	Gene (Genbank)	Subcellular Location	Domains (Accession)
Q8XKM6	LTD domain-containing protein	190 109	CPE1368	Cell wall-anchored	Lamin-tail domain (LTD) (pfam00932)<br>Nuclease COG2374 super family (cl28586)<br>DUF5689 super family (cl40945)<br>CehA/McbA family metallohydrolase (cl41623)<br>ABC-transport superfamily (cl23805)<br>YhcR_OBF_like (cd04486)<br>PRK12366 super family (cl36118)<br>LPXTG cell wall anchor (TIGR01167)
Q8XHG5	Hydrolase, TatD family	29 592	CPE2520	Cytosolic	TatD like proteins (cd01310)
Q8XI04	HNH endonuclease	15 780	CPE2320	Cytosolic	HNHc endonuclease (cd00085)
A0A6M1VU75	SH3 domain-containing protein	61 116	NGT59364.1	Secreted	Pneumococcal surface protein A (cl41532);<br>Bacterial SH3 domain (pfam08239)<br>Pre-toxin TG (cl16891)<br>HNHc endonuclease (cd00085)
R7FRH6	Putative DNase/RNase endonuclease	51 824	CDE16297.1	Secreted	Staphylococcal nuclease homologue (cl00140)
A0A353J425	Endonuclease	28 204	HBF66260.1	Secreted	Endonuclease_NS (smart00892)
H2JJC9	DNA/RNA endonuclease G, NUC1	50 413	AEY64851	Secreted	Lamin-tail domain (LTD) (pfam00932)<br>DNA/RNA endonuclease G, NUC1 (cl30531)
