# Supplementary material for: Clostridium perfringens phospholipase C, an archetypal bacterial virulence factor, induces the formation of extracellular traps by human neutrophils
Source: Front Cell Infect Microbiol. 2023 Oct 27;13:1278718. doi: 10.3389/fcimb.2023.1278718 (PMC10641792; doi:10.3389/fcimb.2023.1278718)
Supplement: Supplementary Table 2 — Q8XKM6 peptides detected from bands 1-17 digestion. [file Table_2.pdf]

Supplementary Table 2

Peptide	Mass	Length	m/z	Accession	PTM
SGVDFGAITK	993,51	10	497,76	Q8XKM6	
QVYSSEDK	954,43	8	478,22	Q8XKM6	
ANNGEN(+.98)EVLLK	1200,60	11	601,31	Q8XKM6	Deamidation (NQ)
AN(+.98)NGENEVLLK	1200,60	11	601,31	Q8XKM6	Deamidation (NQ)
ATISINGTDK	1018,53	10	510,27	Q8XKM6	
SGVDFGAITKGN(+.98)K	1293,66	13	647,82	Q8XKM6	Deamidation (NQ)
TVVDLEAK	873,48	8	437,75	Q8XKM6	
RPVISGEFIDNSGVN(+.98)VESVK	2146,09	20	1074,04	Q8XKM6	Deamidation (NQ)
YEGSPVSQ(+.98)SLEDK	1438,65	13	720,34	Q8XKM6	Deamidation (NQ)
VKGRVEAYQGE	1234,63	11	618,32	Q8XKM6	
TNAESKSNSKVTK	1392,72	13	697,37	Q8XKM6	
ISINGTDK	846,44	8	424,23	Q8XKM6	
YEVNEGQ(+.98)Q(+.98)FR	1270,55	10	636,28	Q8XKM6	Deamidation (NQ)
N(+.98)TVVDLEAKTLTQ(+.98)G	1489,75	14	745,88	Q8XKM6	Deamidation (NQ)
SVQWDVDTENLYDDLGK	1995,91	17	499,99	Q8XKM6	
GQ(+.98)QFRLMLNK	1234,65	10	618,32	Q8XKM6	Deamidation (NQ)
ANGGVEVISK	972,52	10	487,27	Q8XKM6	
ATISINGTDKTFTKSSK	1797,95	17	600,32	Q8XKM6	
VKGRVEAYQG	1105,59	10	553,79	Q8XKM6	
TNAQN(+.98)ISDK	990,46	9	496,24	Q8XKM6	Deamidation (NQ)
TDKDMLLVGDEAK	1433,71	13	478,91	Q8XKM6	
AIEIFNGTGEG	1106,52	11	554,27	Q8XKM6	
NLGEEVTTR	1017,51	9	509,77	Q8XKM6	
ESSITWQPER	1231,58	10	411,54	Q8XKM6	
EPKYEVYK	1054,53	8	528,27	Q8XKM6	
N(+.98)VLPKEN(+.98)SSVGEN(+.98)R	1544,73	14	773,37	Q8XKM6	Deamidation (NQ)
NTVVDLEAK	987,52	9	494,77	Q8XKM6	
TNAQ(+.98)N(+.98)ISDK	991,45	9	496,73	Q8XKM6	Deamidation (NQ)
FTEVINDLENKLGVGSQ(+.98)	1862,93	17	932,48	Q8XKM6	Deamidation (NQ)
SGM(+15.99)EYYIEASDGK	1464,61	13	733,31	Q8XKM6	Oxidation (M)
Q(+.98)VYSSEDK	955,41	8	478,71	Q8XKM6	Deamidation (NQ)
ENEKISLLAK	1143,65	10	572,83	Q8XKM6	
LSIEVYNNSSER	1409,68	12	705,84	Q8XKM6	
ISDGTGSLEDAYK	1354,63	13	452,55	Q8XKM6	
IFAN(+.98)N(+.98)GEN(+.98)EVLLK	1462,72	13	732,36	Q8XKM6	Deamidation (NQ)
AQSEAVEPK	957,48	9	479,75	Q8XKM6	
YEGSPVSQSLEDK	1437,66	13	719,84	Q8XKM6	
KNSDM(+15.99)N(+.98)LK	965,45	8	483,73	Q8XKM6	Oxidation (M); Deamidation (NQ)
NTGEIVLQ	872,46	8	437,24	Q8XKM6	
VNVGLNELK	984,56	9	493,29	Q8XKM6	
ESNFDSHYIELK	1480,68	12	494,57	Q8XKM6	
KQVYSSEDK	1082,52	9	542,26	Q8XKM6	
EEKIDSLGANESK	1418,69	13	710,36	Q8XKM6	
WARDEGN(+.98)ADYFAVTDHSN(+.98) WFDN(+.98)DTEAN	3162,24	27	1055,09	Q8XKM6	Deamidation (NQ)
VMIDGAHAN(+.98)Q(+.98)YVTGN(+.98)	1591,68	15	796,84	Q8XKM6	Deamidation (NQ)
ENSSVGEN(+.98)R	991,42	9	496,71	Q8XKM6	Deamidation (NQ)
AWTNAQNISDKYND	1638,73	14	410,69	Q8XKM6	
LDNEDITKRA	1173,60	10	587,80	Q8XKM6	
AN(+.98)N(+.98)GENEVLLK	1201,58	11	601,80	Q8XKM6	Deamidation (NQ)
VYSSEDK	826,37	7	414,19	Q8XKM6	

QDNHKGK	825,41	7	413,71	Q8XKM6	
VVN(+.98)LIEVGN(+.98)GEG	1200,59	12	601,30	Q8XKM6	Deamidation (NQ)
